# L718Q mutant EGFR escapes covalent inhibition by stabilizing a non-reactive conformation of the lung cancer drug osimertinib†
†Electronic supplementary information (ESI) available: p*K*_a_ shift for Cys797; geometries of TSs identified with QM/MM calculations; analysis of the minimum free-energy path for Cys797 alkylation; analysis of MD replicas; convergence for US simulations; replica of simulation of Cys797 alkylation; conformational FESs obtained from each MD replica. See DOI: 10.1039/c7sc04761d


**DOI:** 10.1039/c7sc04761d

**Published:** 2018-02-12

**Authors:** D. Callegari, K. E. Ranaghan, C. J. Woods, R. Minari, M. Tiseo, M. Mor, A. J. Mulholland, A. Lodola

**Affiliations:** a Department of Food and Drug , University of Parma , Parma , Italy . Email: alessio.lodola@unipr.it; b School of Chemistry , University of Bristol , Bristol , UK; c Medical Oncology Unit , University Hospital of Parma , Parma , Italy

## Abstract

Osimertinib is a third-generation inhibitor approved for the treatment of non-small cell lung cancer. It overcomes resistance to first-generation inhibitors by incorporating an acrylamide group which alkylates Cys797 of EGFR T790M. The mutation of a residue in the P-loop (L718Q) was shown to cause resistance to osimertinib, but the molecular mechanism of this process is unknown. Here, we investigated the inhibitory process for EGFR T790M (susceptible to osimertinib) and EGFR T790M/L718Q (resistant to osimertinib), by modelling the chemical step (*i.e.*, alkylation of Cys797) using QM/MM simulations and the recognition step by MD simulations coupled with free-energy calculations. The calculations indicate that L718Q has a negligible impact on both the activation energy for Cys797 alkylation and the free-energy of binding for the formation of the non-covalent complex. The results show that Gln718 affects the conformational space of the EGFR–osimertinib complex, stabilizing a conformation of acrylamide which prevents reaction with Cys797.

## Abstract 2

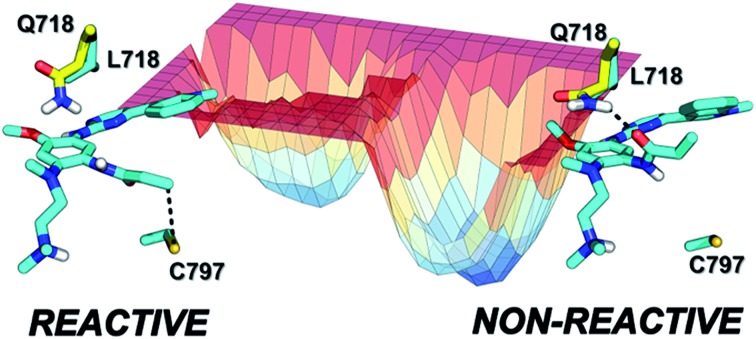
Impact of L718Q mutation on the inhibitory activity of osimertinib on EGFR revealed by free-energy simulations.

## Introduction

**Scheme 1:**
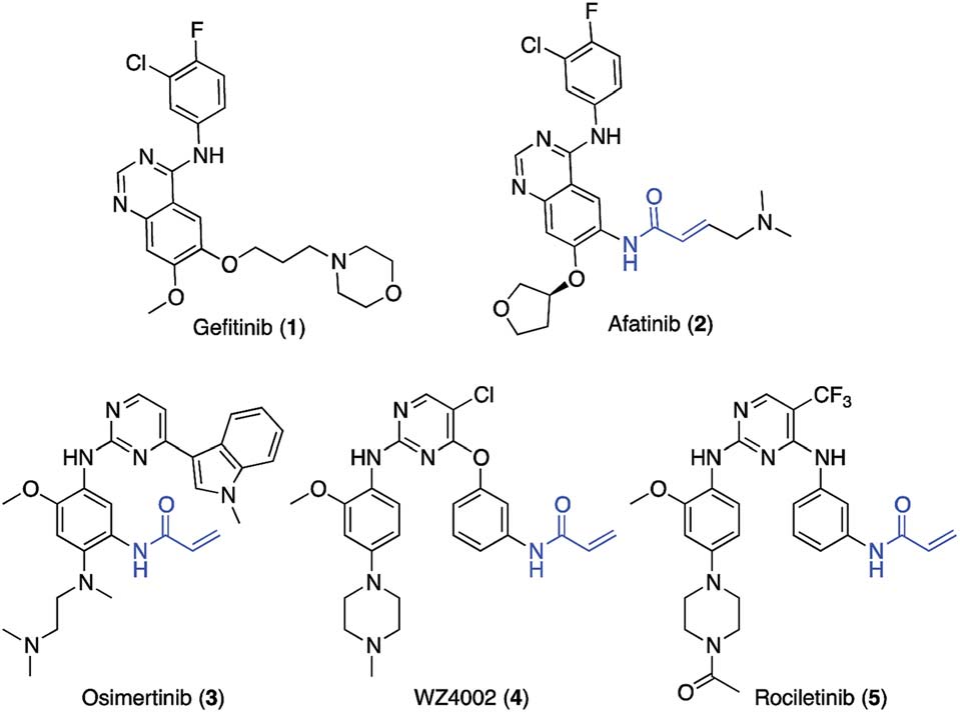
Structures of relevant EGFR inhibitors.

Epidermal growth factor receptor (EGFR) is a transmembrane protein which possesses an extracellular EGF binding domain and an intracellular tyrosine kinase domain.^1^ EGFR activation by its physiological ligand EGF leads to receptor dimerization and phosphorylation, two events that trigger the activation of signal transduction cascades promoting cell proliferation.^2^ In non-small cell lung cancer (NSCLC), overexpression of EGFR or hyper-activating mutations in its kinase domain have been observed in at least 50% of cases.^3^ More than 90% of the known kinase mutations occur as short in-frame deletions in exon 19 or as point mutations in exon 21, the latter resulting in arginine replacing leucine at codon 858 (L858R).^4^ These mutations are critical for NSCLC insurgence and progression as their presence results in constitutive activation of EGFR regardless of the presence of EGF.^5^ First-generation EGFR inhibitors (*e.g.*, gefitinib, **1**, Scheme 1) are widely employed as first-line therapy for NSCLC with activating EGFR mutations (*i.e.* L858R substitutions in exon 21).^6^ Although patients show good responses to the therapy, most of them acquire drug resistance within 1 year treatment, which is driven in about 60% of cases by an additional EGFR T790M point mutation^7^ occurring at the gatekeeper position of the ATP binding site. The change in the steric and lipophilic property of the gatekeeper residue is likely to be responsible for the reduced inhibitory potency of first-generation EGFR inhibitors.^8^,^9^ The second generation of EGFR inhibitors, such as afatinib (**2**, Scheme 1), demonstrated promising activity against T790M-positive tumors in preclinical models.^10^ Due to the presence of an acrylamide warhead capable of alkylating Cys797,^11^ afatinib can circumvent ATP competition and thus overcome the unfavorable effect caused by the presence of methionine at the gatekeeper position.^11^ Nevertheless, in the clinic afatinib showed dose-limiting toxicity resulting from potent inhibition of the wild-type (wt) form of EGFR.^12^

Osimertinib (**3**, Scheme 1)^13^ is a third-generation EGFR inhibitor approved for patients affected by metastatic EGFR T790M mutation-positive NSCLC, who have progressed on or after the therapy with first- and second-generation EGFR inhibitors.^14^

Like other third-generation inhibitors, such as WZ4002 (**4**) and rociletinib (**5**), osimertinib possesses a 2-aminopyrimidine scaffold which confers selectivity for the oncogenic forms of EGFR *versus* the wt, and an acrylamide group that alkylates Cys797 ensuring the ability to potently inhibit EGFR also in the presence of the T790M mutation.^15^

**Fig. 1:**
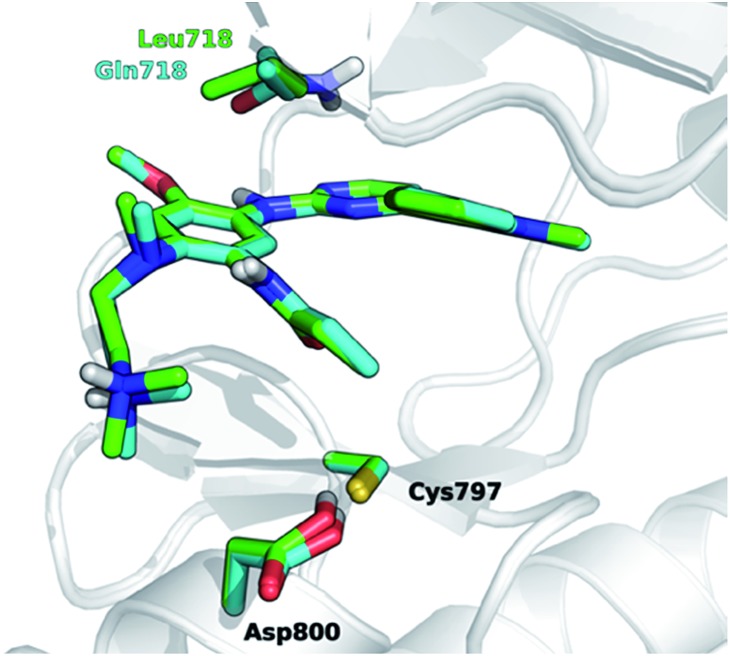
Superposition of 3D models, derived from the X-ray structure 4ZAU.pdb,^20^ representing osimertinib in non-covalent complexes with EGFR T790M (green carbon atoms) and EGFR T790M/L718Q mutants (cyan carbon atoms), respectively.

The development of novel forms of resistance is currently limiting the clinical therapeutic benefit of osimertinib.^16^ The C797S mutation, which replaces the cysteine with the less nucleophilic serine, has emerged as the main determinant of resistance to third generation EGFR inhibitors.^17^ Other mutations, *i.e.*, L718Q occurring in the P-loop and L844V, occurring in the ATP binding site, were initially reported to confer resistance to pyrimidine-based inhibitors, including WZ4002 and rociletinib, *in vitro*.^18^ Moderate activity on cells harboring these mutations was maintained by osimertinib, suggesting that it might be still clinically effective in patients harboring L718Q or L844V mutation. However, a cell proliferation assay conducted in Ba/F3 cells expressing L858R/T790M/L718Q EGFR mutant, showed that osimertinib was considerably less potent (∼100-fold) at inhibiting cell growth than in Ba/F3 cells expressing either L858R/T790M double mutant or L858R/T790M/L844V triple mutant. This suggests that L718Q mutation likely affects the ability of osimertinib to irreversibly inhibit EGFR. As a matter of fact, clinical resistance to osimertinib has recently been reported in a NSCLC patient expressing EGFR L858R/T790M who also acquired the L718Q mutation.^19^ L718Q thus emerges as a mutation able to reduce osimertinib potency *in vitro* and to confer NSCLC resistance *in vivo*.^19^ Starting from the visual inspection of the X-ray structure of EGFR–osimertinib complex,^20^ it has been proposed that the replacement of a leucine with a glutamine at the position 718 of EGFR could reduce the affinity of EGFR for osimertinib and/or hinder Cys797 alkylation by the acrylamide warhead.^19^ Considering that Gln718 occupies a peripheral position of the ATP binding site of EGFR (Fig. 1), the precise role of Gln718 in reducing osimertinib activity remains largely unexplained.

Computational simulation protocols able to rationalize the effects of a mutation in the binding site of EGFR have the potential to contribute significantly to anti-cancer drug discovery:^21^ such methods should help in the design of novel inhibitors able to circumvent mutations. Given the increasing interest in and clinical importance of covalent inhibitors, there is a particular need for methods able to analyze effects of mutation on covalent inhibition.

In the present work, we investigate the impact of L718Q mutation on the inhibitory activity of osimertinib by modelling both its chemical step (*i.e.*, Cys797 alkylation) and recognition step (*i.e.* formation of the non-covalent, pre-reactive EGFR–osimertinib complex). We started from X-ray^20^ derived models of osimertinib in complex with EGFR T790M and EGFR T790M/L718Q mutants. We assessed the influence of the L718Q mutation in EGFR inhibition, estimating for the two molecular systems: (i) the preferred ionization state for Cys797; (ii) the energetics for Cys797 alkylation; (iii) the free-energy of binding for the formation of the non-covalent complex; (iv) the conformational space explored by osimertinib within the two considered EGFR variants. An array of diverse and complementary computational methods was applied. These include molecular dynamics (MD) simulations,^22^ coupled with umbrella sampling (US),^23^ hybrid quantum mechanics molecular mechanics (QM/MM)^24^ and replica-exchange/thermodynamic integration (RETI)^25^ approaches.

## Results and discussion

### Ionization state of Cys797

**Scheme 2:**
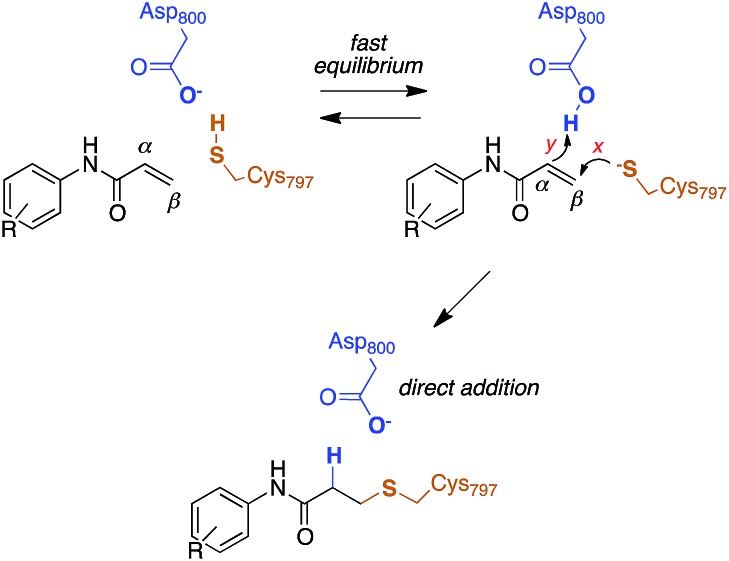
Reaction mechanism of Cys797 alkylation for acrylamide inhibitors of EGFR.

The reactivity of cysteines with electrophilic compounds depends first of all on the protonation state of their thiol group, as indicated by *e.g.*, the pH dependence of the reaction rate for covalent bond formation.^26^ EGFR has a solvent-exposed cysteine (Cys797) that is the site of covalent modification by acrylamide-containing irreversible EGFR inhibitors.^6^,^27^ The Cys797 thiol is not only readily alkylated by Michael acceptors, but is also easily transformed into sulfenic acid (*i.e.*, Cys-S-OH) in the presence of oxidative stimuli.^27^ This evidence to date supports the hypothesis that the Cys797 thiol exists in the anionic form at physiological pH. This could be due to the presence of an organized microenvironment^28^ in EGFR able to stabilize its negative charge.^29^ Furthermore, the Cys797 side chain makes contacts with Asp800, with the sulfur atom at a short distance from one of the oxygen of the carboxylic group, *i.e.*, 3.3 Å in the EGFR–osimertinib structure.^20^ Thus, the carboxylate function of Asp800 is probably the natural acceptor of the Cys-SH proton (Scheme 2).

Previous free-energy simulations have shown that, in the presence of a *N*-(4-anilinoquinazolin-6-yl)acrylamide inhibitor, Cys797–S^–^/Asp800–COOH pair is significantly more stable than Cys797–SH/Asp800–COO^–^.^30^ Moreover, titration experiments on wt-EGFR showed that Cys797 has a p*K*_a_ of 5.5,^31^ a value significantly lower than that of a free thiol in solution (≈8.0), and approaching that of a carboxylic acid. This supports the computational finding that the most abundant protonation state for this cysteine/aspartate pair of EGFR is Cys797–S^–^/Asp800–COOH. Therefore, this was the protonation state employed in our models; we also investigated p*K*_a_ shifts of Cys797 and modeled the proton transfer between it and Asp800.

We began by evaluating the impact of the L718Q mutation on the p*K*_a_ of Cys797. With this aim, we built molecular models of EGFR T790M and T790M/L718Q mutants using the X-ray structure of osimertinib in non-covalent complex with wt-EGFR.^20^ The models were equilibrated by molecular dynamics (MD) simulations (see Experimental section for details). For multiple snapshots taken from the MD simulations, p*K*_a_ calculations performed with PropKa (which uses an empirical scoring function)^32^ and H++ (which solves the Poisson–Boltzmann equation)^33^ indicated that L718Q mutation has no significant effect on the acidity of Cys797 thiol group, with negligible p*K*_a_ shifts calculated by both methods (0.02 ± 0.38 for PropKa and 0.47 ± 0.43 for H++, respectively; see also Table S1, ESI†). It should be pointed out that these Δp*K*_a_ values are simple empirical estimates and thus they may suffer from limited accuracy.

We next investigated if the L718Q mutation could affect the prototropic equilibrium of the Cys797/Asp800 pair. Applying a SCC-DFTB/AMBER99SB QM/MM potential^34^ with umbrella sampling (US) MD simulations,^23^ we calculated the free-energy difference between the Cys797–S^–^/Asp800–COOH and Cys797–SH/Asp800–COO^–^ states. Starting from the molecular models of osimertinib in complex with EGFR T790M or the EGFR T790M/L718Q variant, the proton present on the carboxylic group of Asp800 was moved toward the thiolate group of Cys797 in US MD (see Experimental section).

SCC-DFTB/AMBER99SB QM/MM US MD simulations showed that the Cys797–S^–^/Asp800–COOH state was of similar stability to the Cys797–SH/Asp800–COO^–^ state for EGFR T790M (estimated difference of 0.5 ± 0.2 kcal mol^–1^), while in the case of EGFR T790M/L718Q, the Cys797–S^–^/Asp800–COOH state was more stable by 2.0 ± 0.3 kcal mol^–1^. It appears that the L718Q mutation actually stabilizes the thiolate form of Cys797. It is apparent that the effects of this mutation on inhibition by osimertinib alkylation are not caused by an increase in the p*K*_a_ of Cys797.

### Reaction energetics for Cys797 alkylation

Computational studies recently performed by us^30^ and others^35^ indicate that acrylamide-based inhibitors of EGFR alkylate Cys797 by a direct addition mechanism in which the thiolate group of Cys797 attacks the β carbon of the warhead, while the carboxylic acid group of Asp800 protonates the α carbon, leading to a stable 3-(alkylsulfanyl)propanamide adduct (Scheme 2). Using this reaction scheme, we modelled the Michael-type addition of Cys797 to osimertinib, for both EGFR T790M and EGFR T790M/L718Q mutants.

To analyse the reaction energetics, the free-energy surface (FES) of the direct addition mechanism was calculated from US simulations at the SCC-DFTB/AMBER99SB level, using simple reaction coordinates calculated from distances between the reactive atoms: S_Cys797_–Cβ_acrylamide_ distance modeled the nucleophilic attack (event ***x***), and the difference of distances [(H_Asp800_–Cα_acrylamide_) – (H_Asp800_–O_Asp800_)] described the protonation at Cα of acrylamide (event ***y***). While the SCC-DFTB method is known to underestimate absolute barriers in many cases, this approach has been satisfactorily applied to elucidate the effects of mutations on several enzyme-catalyzed reactions; it provides useful and predictive relative barriers.^36^

**Fig. 2:**
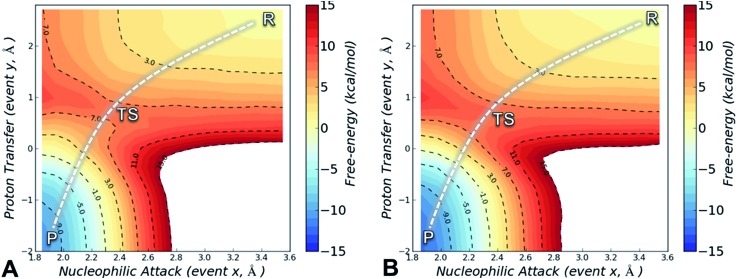
Free-energy surfaces for Cys797 alkylation by osimertinib in EGFR T790M (panel A) and in EGFR T790M/L718Q (panel B), calculated from umbrella sampling simulations at the SCC-DFTB/AMBER99SB level of QM/MM theory. The reaction coordinates (nucleophilic attack and proton transfer), are given in Å. Free energies are given in kcal mol^–1^, and the contour levels are set at 1 kcal mol^–1^ while dashed-contour lines are set every 4 kcal mol^–1^.

The FES is a two-dimensional projection of a chemical process that involves simultaneous changes in two coordinates. Analysis of the FES is useful to explain how changes in the microenvironment of the enzyme affect the position and geometry of the transition state (TS), and thus the energy barrier of the investigated process.^37^ The SCC-DFTB/AMBER99SB FES for the alkylation of Cys797 by osimertinib for EGFR T790M is shown in Fig. 2A, and that for EGFR T790M/L718Q in Fig. 2B. In both cases, the minimum free-energy path connecting reactants (***R***, upper right corner) and the products (***P***, bottom left corner) follows a diagonal pathway, indicating that nucleophilic attack and protonation of the incipient carbanion species are tightly coupled events. No carbanion/enolate species was identified as a stable minimum, consistent with previous DFT calculations on the addition of thiolates to acrylamides.^30^,^35^

The activation free-energy (Δ*A*_act_) at the SCC-DFTB/AMBER99SB level of theory is 7.8 ± 0.2 kcal mol^–1^ for alkylation of EGFR T790M and of 8.1 ± 0.2 kcal mol^–1^ for alkylation of EGFR T790M/L718Q. The barrier to the forward reaction (formation of the covalent complex) is unaffected by the L718Q mutation. The reaction free-energy (Δ*A*_reac_) is highly negative: –10.3 ± 0.2 and –12.2 ± 0.2 kcal mol^–1^ for EGFR T790M and EGFR T790M/L718Q, respectively, indicating that Cys797 alkylation by osimertinib is highly exergonic and thus a spontaneous process. The covalent adduct (products) is predicted to be slightly more stable in the L718Q mutant, thus the barrier to the reverse reaction is a little higher, but this is not expected to be significant as covalent inhibition is likely to be effectively irreversible. Overall, these results indicate that L718Q mutation does not significantly change the energetics of the alkylation. Consistent with this finding, analysis of the geometries identified along the minimum free-energy path connecting ***R*** to ***P*** indicates that L718Q mutation has a negligible impact on the TS geometries obtained by US simulation (Table S2†).

**Fig. 3:**
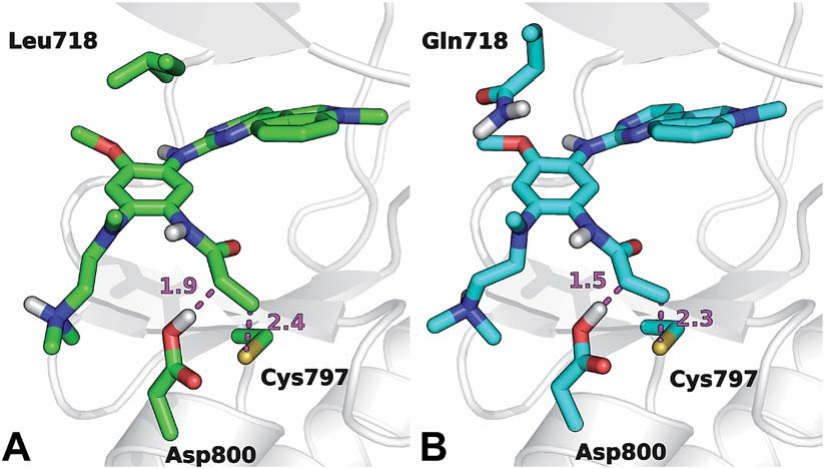
Representative structures of the TS for Cys797 alkylation in the case of EGFR T790M (panel A, green carbon atoms) and EGFR T790M/L718Q (panel B, cyan carbon atoms). Important distances (in Å) are shown by dashed magenta lines.

The conformation adopted by the two conjugated double bonds of the acrylamide fragment, which remained s-*cis* during the entire alkylation process, is similar in the two reaction paths. Further analysis of the minimum free-energy paths (Fig. S1 and S2†), revealed that for both systems the key event of the reaction was the nucleophilic attack of the Cys797 sulfur atom on the acrylamide Cβ, which required complete desolvation of the thiolate anion. In agreement with this finding, structures of the TS for Cys797 alkylation of EGFR T790M, show that the formation of S–Cβ bond is quite advanced, with an average (S–Cβ) distance of 2.41 ± 0.05 Å. In contrast, protonation of the Cα by Asp800 was not very advanced at the TS, with an average H–Cα distance of 1.85 ± 0.08 Å (Fig. 3A). In the case of the EGFR T790M/L718Q variant, the TS structures were slightly more advanced toward the products, as indicated by S–Cβ and H–Cα distances of 2.25 ± 0.07 Å and 1.48 ± 0.04 Å, respectively (Fig. 3B). These minor differences in the average geometries of the TS for the direct addition mechanism explain the negligible difference (0.3 kcal mol^–1^) in the computed activation free-energies Δ*A*_act_. Similar geometries were also found for the products of the reaction.

The thioether adduct ***P*** was characterized by S–Cβ and H–Cα distances of 1.83 ± 0.03 Å and 1.11 ± 0.04 Å, respectively for EGFR T790M. The corresponding S–Cβ and H–Cα distances were 1.84 ± 0.02 Å and 1.10 ± 0.03 Å for the T790M/L718Q variant, respectively. Thus, analysis of reaction paths shows no significant difference for the two EGFR mutants. All the simulation evidence thus indicates that the L718Q mutation does not reduce the reactivity of Cys797. It is important to note that the starting point (reactant/non-covalent complex) for the QM/MM calculations is a reactive conformation in which the nucleophilic sulfur is in close proximity to the electrophile; these simulations therefore do not address possible changes in favored conformations in the non-covalent complex, which are investigated below.

### Binding affinity for the formation of osimertinib/EGFR mutant non-covalent complexes

It has been recently proposed that the main effect of the replacement of Leu718 with Gln718 could be to disrupt the beneficial hydrophobic/steric interactions involving the methoxyphenyl moiety^38^ of third generation EGFR inhibitors (Fig. 1) and thus to hamper the formation of the non-covalent complex through a significant reduction of inhibitor affinity. We tested this hypothesis *in silico* by calculating the binding free-energy (Δ*A*_bind_) of osimertinib for EGFR T790M and EGFR T790M/L718Q, using the WaterSwap method^39^ in combination with conventional MM MD simulations (see Experimental section) performed using the AMBER99SB force field.^40^ WaterSwap estimates Δ*A*_bind_ using a replica-exchange thermodynamic integration (RETI) algorithm along a reaction coordinate that switches the ligand of interest with an equivalent volume of water in the protein binding site.^39^ While this methodology tends to overestimate the values of Δ*A*_bind_, it is able to capture the effect of mutations occurring in the binding site or in its surroundings.^41^ To obtain a robust estimate of the binding free-energy, ten independent WaterSwap calculations were performed for each system *i.e.*, starting from ten different protein–ligand structures taken from a 300 ns-long MD simulation. WaterSwap simulations indicate (Table S3†) that osimertinib has similar Δ*A*_bind_ for EGFR T790M (–34.9 ± 0.7 kcal mol^–1^) and EGFR T790M/L718Q (–34.8 ± 1.9 kcal mol^–1^) suggesting that L718Q mutation does not significantly affect inhibitor affinity.

### Dynamics of osimertinib/EGFR mutant complexes

We finally investigated whether L718Q mutation affects the conformational space of osimertinib in the EGFR mutant–osimertinib complexes. We performed extensive MD simulations (4 independent replicas of 300 ns each) of osimertinib in non-covalent complex with EGFR T790M and with EGFR T790M/L718Q, using the AMBER99SB force field^40^ (see Experimental section for details). We then analyzed the simulations to assess the influence of the mutation on: (i) the binding of the 2-aminopyrimidine scaffold of osimertinib to EGFR hinge region within the ATP binding pocket, and (ii) the accommodation of the acrylamide warhead.

Analysis of the trajectories indicated that L718Q mutation has only a small effect on the overall stability of the EGFR protein. The root-mean square deviation (RMSD) of the backbone atoms for the first set of 300 ns of MD simulation was of 1.60 ± 0.49 Å for EGFR T790M and 2.20 ± 0.31 Å for EGFR T790M/L718Q. On the other hand, a significant increase in the flexibility of the P-loop, *i.e.* the glycine-rich stretch where the mutation is located, was observed when Leu718 was replaced by Gln. Backbone-atom RMSD values were 1.99 ± 0.49 Å and 3.50 ± 0.85 Å for EGFR T790M and EGFR T790M/L718Q, respectively. However, this increased flexibility of the P-loop did not affect the overall stability of the EGFR–osimertinib complex: the RMSD for the heterocyclic core of osimertinib was 1.13 ± 0.32 Å for EGFR T790M and 1.62 ± 0.49 Å for the EGFR T790M/L718Q double mutant. Also, the distance of the key H-bond formed by the N3-pyrimidine nitrogen of osimertinib and the backbone N–H group of Met793 remains essentially the same *i.e.*, 2.19 ± 0.21 Å for EGFR T790M and 2.11 ± 0.15 Å for EGFR T790M/L718Q double mutant. Similar behavior was observed in all the simulations (see Fig. S3–S6, ESI†).

**Fig. 4:**
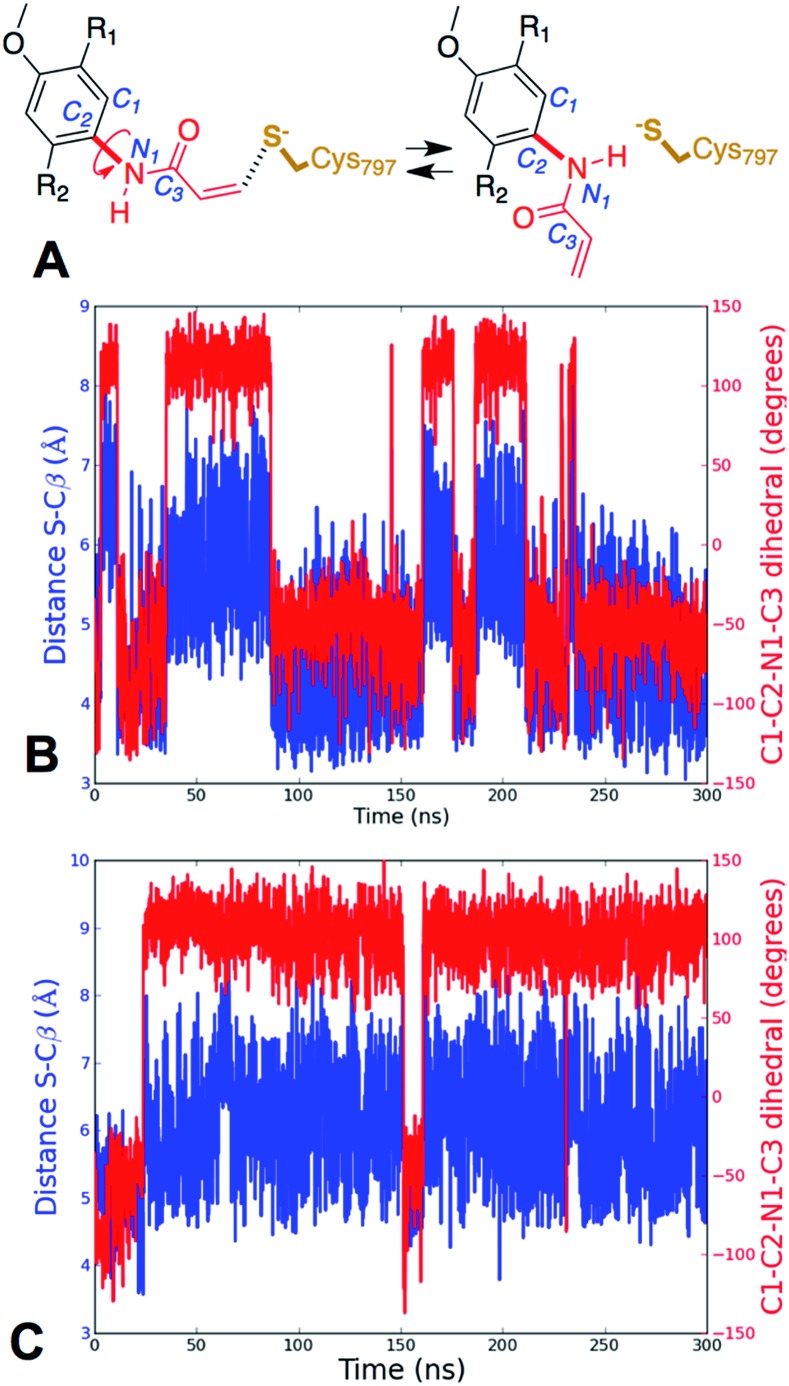
Definition of C_1_–C_2_–N_1_–C_3_ dihedral angle (panel A), and time series for the S–Cβ distance (blue line) and the C_1_–C_2_–N_1_–C_3_ dihedral (red line) for EGFR T790M (panel B) and EGFR T790M/L718Q (panel C).

Further analysis of the MD simulations showed that the presence of Gln instead of Leu did not dramatically alter the average distance between Cys797 sulfur atom and the Cβ of the acrylamide, which moves from 4.9 ± 0.90 Å for EGFR T790M to 6.0 ± 1.0 Å for EGFR T790M/L718Q. However, detailed analysis of the MD trajectories revealed that for EGFR T790M, the fraction of reactive conformations, *i.e.* configurations in which the nucleophile (Cys797 S) and electrophile (acrylamide Cβ) are separated by a distance smaller than the sum of their van der Waals radii^42^ (*i.e.* 3.9 Å with the AMBER99SB force field), corresponds to 20.5% of structures, while in the case of the EGFR T790M/L718Q double mutant, only 0.7% of structures have Cys797 sulfur and acrylamide Cβ at distances shorter than 3.9 Å. Similar results were obtained in other MD replicas (Table S4†). In these trajectories, the distribution of S–Cβ distances depends on the values assumed by the rotatable bond connecting the nitrogen of the acrylamide to the phenyl ring of osimertinib (Fig. 4A). Analysis of the time series of S–Cβ distance and C_1_–C_2_–N_1_–C_3_ dihedral angle (which describes the rotation along the above-mentioned C–N bond) indicates that these two geometrical descriptors are highly correlated (Fig. 4). When the S–Cβ distance approached the critical value of 3.9 Å, the dihedral angle assumed values of –55° ± 20°, consistent with the value observed in the X-ray structure, while when S–Cβ distance overcame the value of 5.5 Å, a nearly 180° shift in the C_1_–C_2_–N_1_–C_3_ dihedral angle was observed.

This trend was observed both in EGFR T790M (Fig. 4B) and in EGFR T790M/L718Q (Fig. 4C). The time series reported in Fig. 4B and C show the existence of two conformational states for EGFR–osimertinib non-covalent complexes. Similar trends were also observed when the MD simulations were replicated (Fig. S7†).

**Fig. 5:**
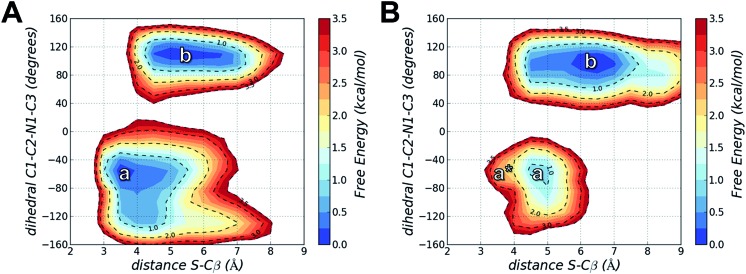
Free energy surfaces calculated from the frequency distribution of conformations obtained from four independent 300 ns MD simulations of EGFR T790M (panel A) and EGFR T790M/L718Q (panel B) in non-covalent complex with osimertinib. ***a*** and ***b*** identify two conformational basins in the space of S–Cβ distance and C_1_–C_2_–N_1_–C_3_ dihedral angle; a* represents, in panel B, the region of reactive conformations for EGFR T790M/L718Q–osimertinib (which approximately corresponding to basin ***a*** in **A**). Free energies are given in kcal mol^–1^, the contour levels are set at 0.25 kcal mol^–1^ while dashed-contour lines are set every 1 kcal mol^–1^ with an additional line at 3.5 kcal mol^–1^.

Using MD simulations of the T790M and T790M/L718Q systems, we calculated conformational FESs along S–Cβ distance and the C_1_–C_2_–N_1_–C_3_ dihedral angle. These FESs were built by pooling the snapshots collected from four MD replicas each of 300 ns (*i.e.* from a total of 1.2 μs MD simulations for each system) and then performing a histogram analysis of the conformational frequencies (see Experimental section). The resulting FESs confirmed the existence of two basins (labelled as ***a*** and ***b***) for both EGFR T790M (Fig. 5A) and EGFR T790M/L718Q (Fig. 5B) mutants.

In the case of EGFR T790M, the region ***a*** identifies a well-defined minimum centered at a S–Cβ distance of 3.5 Å and a C_1_–C_2_–N_1_–C_3_ dihedral angle of –55°. This corresponds to the geometry of the EGFR T790M–osimertinib complex belonging to basin ***R*** of Fig. 2. Region ***a*** thus contains reactive conformations, *i.e.*, conformations extending from the ground state of the complex that lie on the transition path leading to the alkylation of Cys797. Region ***b*** identified a wider minimum corresponding to unreactive conformations of the EGFR–osimertinib complex, in which the S–Cβ distance was dispersed over a 4.5–6.5 Å interval due to a change in C_1_–C_2_–N_1_–C_3_ dihedral angle, which adopted conformations in the range +100° to +120°. ***a*** and ***b*** possess nearly the same free-energy and thus they can be regarded as equally populated conformational states.

**Fig. 6:**
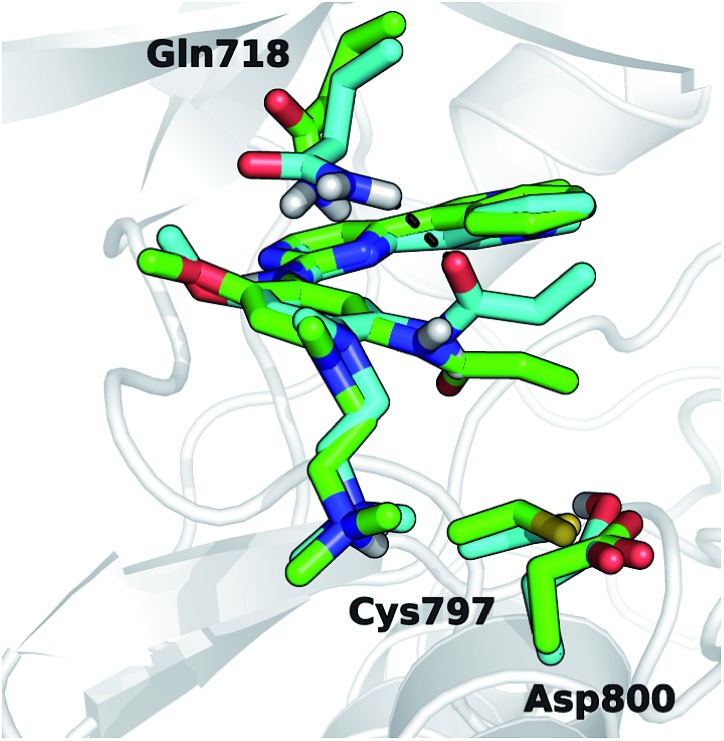
Representative geometry structures for EGFR T790M/L718Q–osimertinib complexes taken from basin a* (green carbon atoms) and ***b*** (cyan carbon atoms) of Fig. 5B. The H-bond involving Gln718 and the acrylamide carbonyl is depicted with a dashed black line.

For EGFR T790M/L718Q, significant changes were observed in the positions of minima ***a*** and ***b***, and in their relative populations. Firstly, minimum ***a*** does not correspond to any reactive conformations. Configurations in this area of the FES feature a S–Cβ distance approaching 4.5 Å and thus are hardly capable of leading to Cys797 alkylation. Minimum ***b*** describes unreactive conformations where the S–Cβ distance is even higher (5.5–7.0 Å range) while the C_1_–C_2_–N_1_–C_3_ dihedral angle remains close to the interval ranging from +90° to +110°, already observed for the unreactive basin of the T790M mutant. Importantly, basin ***b*** represents the global minimum of the computed FES for the EGFR T790M/L718Q system, being more stable than basin ***a*** by nearly 1 kcal mol^–1^. It is still possible to identify reactive geometries for the EGFR T790M/L718Q mutant. These conformations are situated on a region of the FES, labelled with a* (Fig. 5B), which does not correspond to a free-energy minimum. a* is higher in energy than basin ***b*** by ≈3 kcal mol^–1^. The stabilization of basin ***b*** compared to ***a*** and a* in the L718Q mutant arises from a H-bond between the side chain of Gln718 and the carbonyl oxygen of the acrylamide group of osimertinib (Fig. 6). The hydrogen bond is maintained for a significant part of the MD simulations. Comparison of the time evolution for the H-bond distance, the C_1_–C_2_–N_1_–C_3_ dihedral angle (Fig. S8†) and the S–Cβ distance (Fig. S9†), indicates that formation of this H-bond is the key driver leading to stabilization of the unreactive states for EGFR T790M/L718Q–osimertinib complex. The hydrogen bond between the sidechain of Gln718 and the carbonyl oxygen of acrylamide drives the transition of the C_1_–C_2_–N_1_–C_3_ dihedral angle and places the electrophile Cβ far away from the Cys797 thiolate. This H-bond mediated stabilization of a non-reactive conformation; this conformational change of the non-covalent complex will hamper the formation of a covalent bond and thus appears to be the central cause of how the T790M/L718Q mutant escapes irreversible inhibition by osimertinib. These conclusions are supported by all the other MD replicas (which are reported in Fig. S8 and S9 of the ESI†).

## Conclusions

The clinical use of third-generation EGFR inhibitors is revealing the insurgence of novel mutations which confer to lung cancer cells the ability to escape EGFR inhibition. Among recently reported mutations, L718Q has emerged as an intriguing mutation considering that this occurs in a region of EGFR peripheral to the ATP binding site and that its effect is not obvious (in contrast *e.g.* to the C797S mutation). Here, a multilevel computational approach has provided evidence that L718Q mutation does not affect the ionization state of Cys797 (the intrinsic reactivity of Cys797 is not reduced) nor the activation free-energy for the direct addition of acrylamide to the α,β double bond. Free-energy calculations, performed with a replica exchange-thermodynamic integration method, indicate that L718Q mutation does not affect the affinity of osimertinib for the EGFR active site. On the other hand, simulations show that Gln718, through an H-bond with the acrylamide warhead, stabilizes a specific conformation of osimertinib in which the electrophilic Cβ of its acrylamide substituent is kept away from the Cys797 thiolate. This conformation hampers Cys797 alkylation and may thus favor the displacement of osimertinib from EGFR by ATP, which is present in cells at high concentrations.^11^ This global picture rationalizes the poor inhibitory activity of osimertinib in Ba/F3 cells harboring the L718Q mutation^18^ and the lack of therapeutic activity of osimertinib in patients who have acquired this mutation.^19^ We believe that the protocol described here represents a viable and generalizable strategy to investigate *in silico* the effects of mutations on covalent inhibition. This requires consideration of several distinct factors: (i) evaluation of the ionization state of the nucleophile; (ii) estimation of the free-energy barrier for covalent adduct formation; (iii) binding affinity for the formation of the non-covalent complex; (iv) mutual orientation of the reactants within the target active site. Moreover, our conclusion about the conformational selection induced by L718Q mutation should be useful for the design of new EGFR inhibitors overcoming drug resistance.

## Experimental

### Model building and equilibration

EGFR T790M and EGFR T790M/L718Q in complex with osimertinib were built starting from the crystal structure of the non-covalent complex between wt EGFR and osimertinib (4ZAU.pdb),^20^ using the LEaP program available in AMBER 16 (AMBER 2016, University of California, San Francisco, CA). The resulting complexes were immersed in a box of TIP3P water molecules^43^ and neutralized with 3 chloride ions. The total system size was 46 936 atoms (14122 TIP3P waters) in the case of the EGFR T790M–osimertinib complex and 46 283 atoms in the case of the EGFR T790M/L718Q–osimertinib complex (13905 waters). Solvated complexes were energy-minimized, gradually heated to 300 K in NVT ensemble and equilibrated at pressure of 1 atm in NPT ensemble. These systems were then submitted to a short MD simulation (10 ns) in the NVT ensemble. In all these simulations, the AMBER99SB force field was applied to describe protein atoms while the generalized AMBER force field (GAFF)^44^ was employed to describe osimertinib. The pmemd.cuda code of AMBER16 was used to perform these simulations on NVIDIA K20 GPU cards. Full electrostatic and van der Waals interactions were computed within a cutoff of 10 Å and long range electrostatic interactions were treated using the particle mesh Ewald (PME) with 128 × 128 × 128 grid points. Covalent bonds involving hydrogens were constrained using the SHAKE function, allowing the use of a time step of 2 fs. The structures extracted from these MD trajectories (snapshots) were employed to estimate p*K*_a_ for Cys797, for QM/MM simulations, and to perform further MD simulations (see below).

### p*K*_a_ calculations

Ten equally spaced snapshots were taken from the 10 ns MD trajectories for EGFR T790M–osimertinib and EGFR T790M/L718Q–osimertinib complexes. The structures were exported as PDB files and submitted to p*K*_a_ calculations using Propka3.0 (ref. 32) and H++ (ref. 33) software using default settings. Individual Δp*K*_a_ values were calculated as Cys797 p*K*_a_ of EGFR T790M minus Cys797 p*K*_a_ of EGFR T790M/L718Q, while the final values reported in main text resulted from their average. The uncertainty of Δp*K*_a_ was estimated calculating the standard error of the mean (SEM). Absolute p*K*_a_ values are reported in Table S1 in the ESI.†

### Application of the QM/MM potential

QM/MM has been extensively applied to study reaction mechanisms in condensed phase, including enzymes. This approach has the advantage that large systems can be investigated with a reasonable computational cost. The central idea behind the QM/MM approach is the partitioning of the system of interest into two interacting regions: (i) a small QM region, where the chemical bond breaking and forming take place and (ii) a large MM region surrounding the QM atoms. Here, we applied the self-consistent charge-density functional tight binding (SCC-DFTB)^45^ model to describe the QM region and the AMBER99SB force field to describe the MM region. The SCC-DFTB approach is based on the second-order expansion of the total DFT energy with respect to the charge-density variation. Hybrid QM/MM potentials derived from the SCC-DFTB theory have been widely applied to enzyme-catalyzed reactions and often give reasonable descriptions of reaction geometries and energetics.^46^ In the EGFR–osimertinib complexes, side chain atoms of Cys797, Asp800 and the acrylamide portion of the inhibitor were treated with the SCC-DFTB method with dispersion correction. The resulting QM system was composed of 23 atoms, including three link atoms placed along the C–C bond connecting Cβ of the amino acids to their backbone Cα and along the C–N bond connecting the acrylamide nitrogen to the phenyl ring of osimertinib. The adjust_q function was applied to conserve the charge of the systems.

### US simulation for Cys797 deprotonation by Asp800

A set of US simulations was performed by adding a spring constant of *k* = 100 kcal mol^–1^ Å^–2^ along the difference of distances (H_Asp800_–S_Cys797_) – (H_Asp800_–O_Asp800_). This difference was named as reaction coordinate ***r*** and was sampled from 1 to –1 Å with a step-size of 0.1 Å, giving a total of 21 windows. For each window, 50 ps of QM/MM MD simulation at 300 K in NVT conditions were performed for a total of 1 ns of simulation, using the sander.MPI module of AMBER16. All the atoms of the system (including hydrogens) were allowed to move during the simulations, and a time step of 0.2 fs was used to integrate the equation of motion. The PME approach was used to treat the QM/MM long-range electrostatic interactions. The unbiased FES was calculated by using the weighted histogram analysis method (WHAM).^47^ Convergence of the simulations was evaluated calculating the difference in the free-energy between Cys797–S^–^/Asp800–COOH and Cys797–SH/Asp800–COO^–^ states as function of the simulation time. This difference reached a stable value after 40 ps of simulation for each window (Fig. S10†). The free-energy difference between the two ionization states was calculated at different simulation time once convergence was achieved *i.e.*, after 40, 45 and 50 ps. The final energies reported here represent mean values ± standard deviation.

### US simulation for Cys797 alkylation

US MD simulations were carried out adding a spring constant of 100 kcal mol^–1^ Å^–2^ along the reaction coordinate ***x***, described by the S_Cys797_–Cβ_acrylamide_ distance, and reaction coordinate ***y***, described by the difference of distances (H_Asp800_–Cα_acrylamide_) – (H_Asp800_–O_Asp800_). Coordinate ***x*** was sampled from 3.6 to 1.8 Å with a step-size of 0.1 Å for a total of 19 windows while coordinate ***y*** was sampled from 2.8 to –2.0 Å with a step-size of 0.2 Å for a total of 26 windows. Overall the simulated grid is composed of 494 windows. For each one, 50 ps of QM/MM MD simulation at 300 K in NVT conditions were performed for a total of 24.7 ns. The same settings described for Cys797–Asp800 proton transfer were applied. The presence of a normal distribution of the sampled data along ***x*** and ***y*** was verified within each simulated window. The employed spring constant allowed a satisfactory overlapping of the sampled windows, which is critical to obtain a reliable and continuous FES performed for each umbrella. The unbiased FES was calculated by using the weighted histogram analysis method (WHAM).^47^ Convergence of the simulations was evaluated calculating the Δ*A*_act_ and Δ*A*_reac_ as functions of the simulations time. These two outputs reached stable values within 30 ps of QM/MM MD simulation for each window. Average values and standard deviations reported in the main text were calculated from data collected at 30, 40 and 50 ps of simulation for each window. This was the case of both EGFR–osimertinib complexes (Fig. S11†). The US simulations were also repeated to evaluate their reproducibility (Fig. S12†).

### WaterSwap calculations

From a 300 ns trajectory for the EGFR T790M–osimertinib and EGFR T790M/L718Q–osimertinib complexes, 10 equally spaced snapshots were selected and employed for WaterSwap calculations. Binding free energies were calculated using replica-exchange thermodynamic integration over 16λ windows (0.005, 0.071, 0.137, 0.203, 0.269, 0.335, 0.401, 0.467, 0.533, 0.599, 0.665, 0.731, 0.797, 0.863, 0.929, 0.995) over the WaterSwap reaction coordinate. 30 million Monte Carlo moves were performed for each window, with the free-energy gradient averaged over the last 20 million steps. Monte Carlo sampling involved protein residues and water molecules within 15 Å of the center of osimertinib. Simulations used the set A soft-core parameters described in ref. 39, with a 15 Å coulomb and Lennard Jones non-bonded cutoff. This shifted cutoff was used to better account for long-range electrostatics.

Δ*A*_bind_ values reported here are mean values ± the standard error of the mean, as obtained from 10 independent simulation runs.

### Unbiased MD simulations and free energy calculations

Starting from equilibrated systems of EGFR T790M–osimertinib and EGFR T790M/L718Q–osimertinib complexes we performed 4 runs of 300 ns-long MD simulations at 300 K in NVT conditions using the settings described above. Snapshots collected from MD trajectories (30 000 for each replica, for a total of 120 000 for each system) were employed to build the 2D-free-energy surfaces in the space of S–Cβ distance and C_1_–C_2_–N_1_–C_3_ dihedral angle. The free-energy along these variables was computed applying the following equation:*A*(*r*) = –*k*_B_*T* ln *P*(*r*)where *P* is the probability distribution, *k*_B_ is the Boltzmann constant, and *T* is the simulation temperature. The 2D distribution function *P*(*r*) was obtained performing a histogram analysis of conformational frequencies with the cpptraj module^48^ implemented in AMBER16. Snapshots were separated into classes based on the values of the S–Cβ distance and C_1_–C_2_–N_1_–C_3_ dihedral angle. S–Cβ distance ranged from 2.5 to 9.5 Å with a class width of 0.1 Å, while the C_1_–C_2_–N_1_–C_3_ dihedral angle ranged from –160° to +160° with a class width of 15°. FESs in the space of S–Cβ distance and C_1_–C_2_–N_1_–C_3_ dihedral angle for each replica are reported for EGFR T790M–osimertinib and EGFR T790M/L718Q–osimertinib complexes in Fig. S13 and S14, respectively of the ESI† section.

## Conflicts of interest

There are no conflicts to declare.

## Supplementary Material

Supplementary information

## References

[cit1] Hynes N. E., Lane H. A. (2005). Nat. Rev. Cancer.

[cit2] Schlessinger J. (2000). Cell.

[cit3] Normanno N., De Luca A., Bianco C., Strizzi L., Mancino M., Maiello M. R., Carotenuto A., De Feo G., Caponigro F., Salomon D. S. (2006). Gene.

[cit4] Sharma S. V., Bell D. W., Settleman J., Haber D. A. (2007). Nat. Rev. Cancer.

[cit5] Pao W., Chmielecki J. (2010). Nat. Rev. Cancer.

[cit6] Carmi C., Lodola A., Rivara S., Vacondio F., Cavazzoni A., Alfieri R., Ardizzoni A., Petronini P. G., Mor M. (2011). Mini-Rev. Med. Chem..

[cit7] Engelman J. A., Jan̈ne P. A. (2008). Clin. Cancer Res..

[cit8] Michalczyk A., Klüter S., Rode H. B., Simard J. R., Grütter C., Rabiller M., Rauh D. (2008). Bioorg. Med. Chem..

[cit9] Yun C. H., Mengwasser K. E., Toms A. V., Woo M. S., Greulich H., Wong K. K., Meyerson M., Eck M. J. (2008). Proc. Natl. Acad. Sci. U. S. A..

[cit10] Hirsh V. (2011). Future Oncol..

[cit11] Carmi C., Mor M., Petronini P. G., Alfieri R. (2012). Biochem. Pharmacol..

[cit12] Katakami N., Atagi S., Goto K., Hida T., Horai T., Inoue A., Ichinose Y., Koboyashi K., Takeda K., Kiura K., Nishio K., Seki Y., Ebisawa R., Shahidi M., Yamamoto N. (2013). J. Clin. Oncol..

[cit13] Finlay M. R. V., Anderton M., Ashton S., Ballard P., Bethel P. A., Box M. R., Bradbury R. H., Brown S. J., Butterworth S., Campbell A., Chorley C., Colclough N., Cross D. A. E., Currie G. S., Grist M., Hassall L., Hill G. B., James D., James M., Kemmitt P., Klinowska T., Lamont G., Lamont S. G., Martin N., McFarland H. L., Mellor M. J., Orme J. P., Perkins D., Perkins P., Richmond G., Smith P., Ward R. A., Waring M. J., Whittaker D., Wells S., Wrigley G. L. (2014). J. Med. Chem..

[cit14] Ramalingam S. S., Yang J. C., Lee C. K., Kurata T., Kim D. W., John T., Nogami N., Ohe Y., Mann H., Rukazenkov Y., Ghiorghiu S., Stetson D., Markovets A., Barrett J. C., Thress K. S., Jänne P. A. (2017). J. Clin. Oncol..

[cit15] Cross D. A., Ashton S. E., Ghiorghiu S., Eberlein C., Nebhan C. A., Spitzler P. J., Orme J. P., Finlay M. R., Ward R. A., Mellor M. J., Hughes G., Rahi A., Jacobs V. N., Red Brewer M., Ichihara E., Sun J., Jin H., Ballard P., Al-Kadhimi K., Rowlinson R., Klinowska T., Richmond G. H., Cantarini M., Kim D. W., Ranson M. R., Pao W. (2014). Cancer Discovery.

[cit16] Engel J., Lategahn J., Rauh D. (2016). ACS Med. Chem. Lett..

[cit17] Thress K. S., Paweletz C. P., Felip E., Cho B. C., Stetson D., Dougherty B., Lai Z., Markovets A., Vivancos A., Kuang Y., Ercan D., Matthews S. E., Cantarini M., Barrett J. C., Jan̈ne P. A., Oxnard G. R. (2015). Nat. Med..

[cit18] Ercan D., Choi H. G., Yun C. H., Capelletti M., Xie T., Eck M. J., Gray N. S., Jänne P. A. (2015). Clin. Cancer Res..

[cit19] Bersanelli M., Minari R., Bordi P., Gnetti L., Bozzetti C., Squadrilli A., Lagrasta C. A., Bottarelli L., Osipova G., Capelletto E., Mor M., Tiseo M. (2016). J. Thorac. Oncol..

[cit20] Yosaatmadja Y., Silva S., Dickson J. M., Patterson A. V., Smaill J. B., Flanagan J. U., McKeage M. J., Squire C. J. (2015). J. Struct. Biol..

[cit21] Sutto L., Gervasio F. L. (2013). Proc. Natl. Acad. Sci. U. S. A..

[cit22] De Vivo M., Masetti M., Bottegoni G., Cavalli A. (2016). J. Med. Chem..

[cit23] Sinko W., Lindert S., McCammon J. A. (2013). Chem. Biol. Drug Des..

[cit24] Field M. J., Bash P. A., Karplus M. (1990). J. Comput. Chem..

[cit25] Woods C. J., King M. A., Essex J. W. (2003). J. Phys. Chem. B.

[cit26] Berteotti A., Vacondio F., Lodola A., Bassi M., Silva C., Mor M., Cavalli A. (2014). ACS Med. Chem. Lett..

[cit27] Schwartz P. A., Kuzmic P., Solowiej J., Bergqvist S., Bolanos B., Almaden C., Nagata A., Ryan K., Feng J., Dalvie D., Kath J. C., Xu M., Wani R., Murray B. W. (2014). Proc. Natl. Acad. Sci. U. S. A..

[cit28] Madzelan P., Labunska T., Wilson M. A. (2012). FEBS J..

[cit29] Truong T. H., Carroll K. S. (2012). Biochemistry.

[cit30] Capoferri L., Lodola A., Rivara S., Mor M. (2015). J. Chem. Inf. Model..

[cit31] Truong T. H., Ung P. M., Palde P. B., Paulsen C. E., Schlessinger A., Carroll K. S. (2016). Cell Chem. Biol..

[cit32] Olsson M. H., Søndergaard C. R., Rostkowski M., Jensen J. H. (2011). J. Chem. Theory Comput..

[cit33] Anandakrishnan R., Aguilar B., Onufriev A. V. (2012). Nucleic Acids Res..

[cit34] Seabra G. M., Walker R. C., Elstner M., Case D. A., Roitberg A. E. (2007). J. Phys. Chem. A.

[cit35] Paasche A., Schiller M., Schirmeister T., Engels B. (2010). ChemMedChem.

[cit36] Xu D. G., Guo H. (2009). J. Am. Chem. Soc..

[cit37] Jencks W. P. (1972). Chem. Rev..

[cit38] Yan X. E., Zhu S. J., Liang L., Zhao P., Choi H. G., Yun C. H. (2017). Oncotarget.

[cit39] Woods C. J., Malaisree M., Hannongbua S., Mulholland A. J. (2011). J. Chem. Phys..

[cit40] Hornak V., Abel R., Okur A., Strockbine B., Roitberg A., Simmerling C. (2006). Proteins.

[cit41] Woods C. J., Malaisree M., Long B., McIntosh-Smith S., Mulholland A. J. (2013). Sci. Rep..

[cit42] Lonsdale R., Ranaghan K. E., Mulholland A. J. (2010). Chem. Commun..

[cit43] Jorgensen W. L., Chandrasekhar J., Madura J. D., Impey R. W., L Klein M. (1983). J. Chem. Phys..

[cit44] Wang J. M., Wolf R. M., Caldwell J. W., Kollman P. A., Case D. A. (2004). J. Comput. Chem..

[cit45] Elstner M. (2006). Theor. Chem. Acc..

[cit46] Gruden M., Andjeklović L., Jissy A. K., Stepanović S., Zlatar M., Cui Q., Elstner M. (2017). J. Comput. Chem..

[cit47] Kumar S., Rosenberg J. M., Bouzida D., Swendsen R. H., Kollman P. A. (1992). J. Comput. Chem..

[cit48] Roe D. R., Cheatham T. E. (2013). J. Chem. Theory Comput..

