# Supplementary material for: L718Q mutant EGFR escapes covalent inhibition by stabilizing a non-reactive conformation of the lung cancer drug osimertinib
Source: Chem Sci. 2018 Feb 12;9(10):2740–9. doi: 10.1039/c7sc04761d (PMC5911825; doi:10.1039/c7sc04761d)
Supplement: Supplementary file 1 [file SC-009-C7SC04761D-s001.pdf]

Electronic Supplementary Material (ESI) for Chemical Science.
This journal is © The Royal Society of Chemistry 2018

Electronic Supplementary Information (ESI) for

L718Q mutant EGFR escapes covalent inhibition by stabilizing a non-reactive conformation of the lung cancer drug osimertinib

D. Callegari,[a] K. E. Ranaghan,[b] C. J. Woods,[b] R. Minari,[c] M. Tiseo,[c] M. Mor,[a] A. J. Mulholland,[b] and A. Lodola[a]*

[a]Department of Food and Drug, University of Parma, Parma, Italy; [b]School of Chemistry, University of Bristol, Bristol, UK; [c]Medical Oncology Unit, University Hospital of Parma, Italy;

Table of contents

Table S1. Estimated pKa values for the thiol side chain of Cys797 for EGFR T790M and EGFR T790M/L718Q strucures in complex with osimertinib.

Table S2. Geometries of TSs for Cys797 alkylation identified with QM/MM calculations.

Fig. S1. Analysis of the minimum free-energy path of Cys797 alkylation for EGFR T790M.

Fig. S2. Analysis of the minimum free-energy path of Cys797 alkylation for EGFR T790M/L718Q.

Table S3. Absolute binding free energy **(**ΔA_{bind}) of osimertinib calculated with WaterSwap.

Fig. S3. Analysis of MD trajectory of replica 1.

Fig. S4. Analysis of MD trajectory of replica 2.

Fig. S5. Analysis of MD trajectory of replica 3.

Fig. S6. Analysis of MD trajectory of replica 4.

Table S4. Percentage of reactive configurations in each MD trajectory.

Fig. S7. Time series of the S-Cβ distance and the C1-C2-N1-C3 dihedral for EGFR T790M and EGFR T790M/L718Q from replica 2, replica 3 and replica 4.

Fig. S8. Time series comparing the C1-C2-N1-C3 dihedral with the H-bond distance undertaken by Gln718 and the acrylamide group of osimertinib for each of the four MD replicas.

Fig. S9. Time series comparing the S-Cβ distance with H-bond distance undertaken by Gln718 and the acrylamide group of osimertinib from each of the four MD replicas.

Fig. S10. Convergence of the US simulation of Cys797 deprotonation by Asp800.

Fig. S11. Convergence of the US simulation of Cys797 alkylation by osimertinib.

Fig. S12. SCC-DFTB/AMBER FES of the replica of Cys797 alkylation by osimertinib.

Fig. S13. Conformational FESs along S-Cβ distance and the C1-C2-N1-C3 dihedral angle from each of the four MD replicas for EGFR T790M.

Fig. S14. Conformational FESs along S-Cβ distance and the C1-C2-N1-C3 dihedral angle form each of the four MD replicas for EGFR T790M/L718Q.

Table S1. Computational estimation of the pKa of Cys797 thiol side chain within EGFR T790M mutant or EGFR T790M/L718Q double mutant. In both systems, the osimertinib drug occupied the ATP binding site. Calculations were perfomed with PROPKA or H++ software employing 10 evenly spaced snapshots taken from MD simulations.

Snapshot	*EGFR T790M*		*EGFR T790M/L718Q*	
	**Propka**	**H++**	**Propka**	**H++**
1	10.86	10.32	11.11	10.58
2	9.58	9.38	9.67	9.35
3	9.16	8.97	10.93	10.16
4	11.46	11.11	10.65	9.28
5	10.53	10.23	9.54	8.93
6	9.43	9.22	10.84	10.28
7	9.14	9.21	9.27	8.40
8	9.36	8.70	10.52	9.85
9	11.30	10.63	9.54	8.48
10	10.89	10.84	9.45	8.64
mean	**10.17**	**9.86**	**10.15**	**9.39**
sem	**0.29**	**0.27**	**0.23**	**0.25**

Table S2. Geometrical parameters for approximate TS structures for Cys797 alkylation identified with QM/MM calculations. Geomertical values are reported as mean values ± standard deviation.

	**EGFR T790M**	**EGFR T790M/L718Q**
**S-Cβ distance (**Å)	2.41 ± 0.05	2.24 ± 0.07
**S-Cβ-Cα angle (degrees)**	118.60 ± 4.41	114.10 ± 3.74
**H-Cα distance (**Å)	1.85 ± 0.08	1.48 ± 0.04
**n WAT**	1.64 ±0.48	0.77 ± 0.58

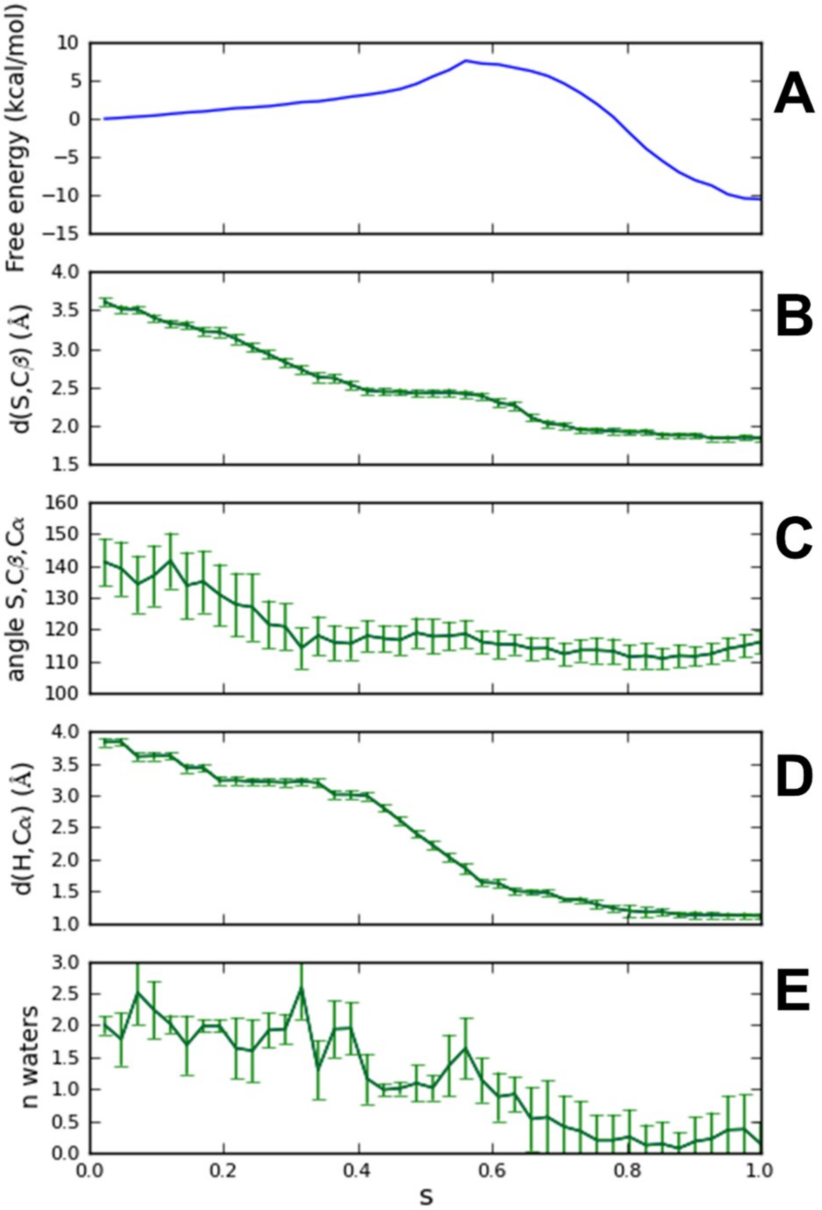

Fig. S1. Analysis of the minimum free-energy path of Cys797 alkylation for EGFR T790M. The variable S identifies a collection of snapshots laying on the minimum free-energy path connecting *R* and *P* (i.e. white line on Figure 2A). Free energy profile of the reaction (Panel A).Evolution of the distance between S_{Cys797} and acrylamide Cß along the path S (Panel B). Evolution of the angle between $S_{Cys797,}$ acrylamide Cβ and acrylamide Cα along S (Panel C). Evolution of the distance between H_{Asp800} and acrylamide Cα along S (Panel D). Number of waters within 3.5 Å of the thiolate S_{Cys797} along S (Panel E). Values are represented as average with error bars representing the standard deviations.

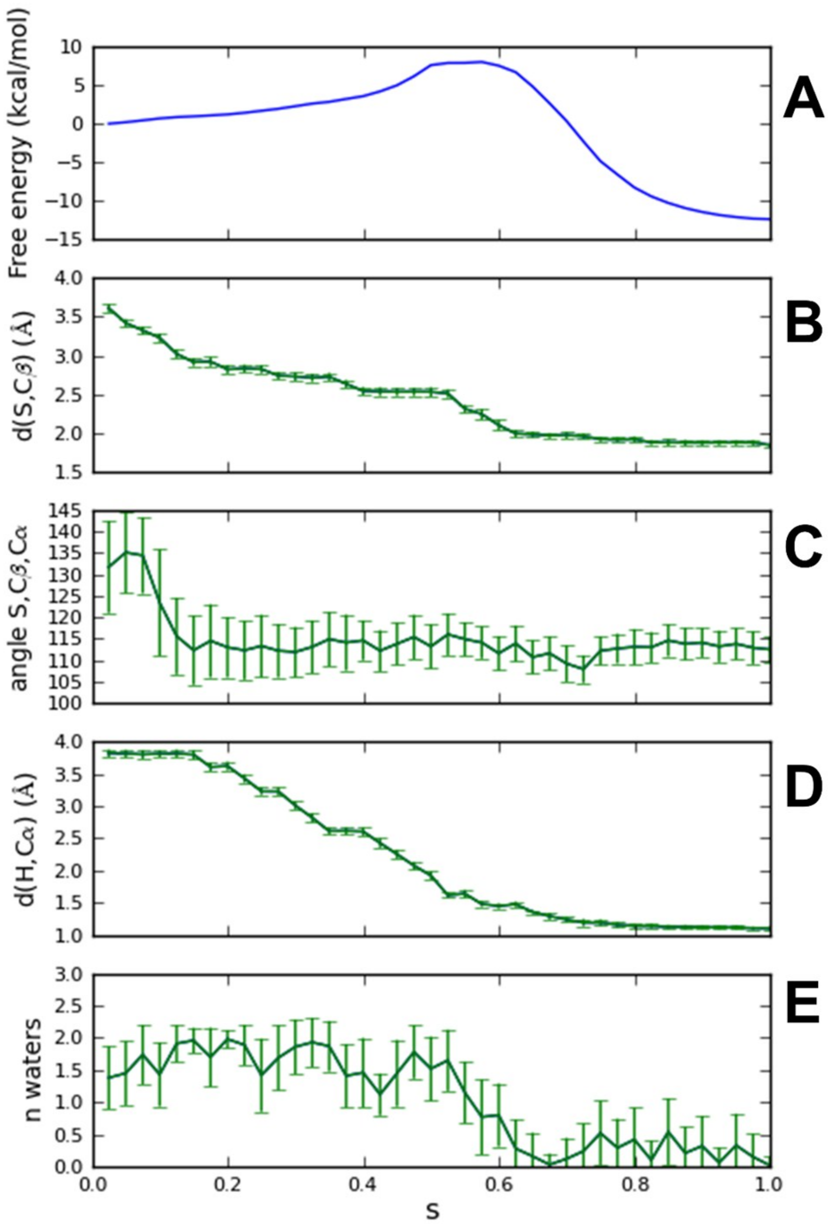

Fig. S2. Analysis of the minimum free-energy path of Cys797 alkylation for EGFR T790M/L718Q. The variable S identifies a collection of snapshots laying on the minimum free-energy path connecting *R* and *P* (i.e. white line on Figure 2B). Free energy profile of the reaction (Panel A). Evolution of the distance between S_{Cys797} and acrylamide Cß along the path S (Panel B). Evolution of the angle between $S_{Cys797,}$ acrylamide Cβ and acrylamide Cα along S (Panel C). Evolution of the distance between H_{Asp800} and acrylamide Cα along S (Panel D). Number of waters within 3.5 Å of the thiolate S_{Cys797} along S (Panel E). Values are represented as average with error bars representing the standard deviations.

Table S3. Absolute binding affinity ($\Delta A_{bind,}$ kcal/mol) of osimertinib for EGFR T790M or EGFR T790M/L718Q estimated with the free-energy perturbation approach *WaterSwap*. Alchemical transformations coupled with RETI calculations were performed starting from 10 distinct snapshots selected from a classical MD trajectory for each molecular system (see main text).

	**EGFR T790M**	**EGFR T790M/L718Q**
**Frame-01**	-30.59	-39.33
**Frame-02**	-36.26	-38.86
**Frame-03**	-36.36	-33.62
**Frame-04**	-31.49	-38.82
**Frame-05**	-35.63	-33.65
**Frame-06**	-34.08	-39.98
**Frame-07**	-35.80	-41.94
**Frame-08**	-35.67	-29.61
**Frame-09**	-35.77	-24.13
**Frame-10**	-37.01	-27.87
**mean**	**-34.87**	**-34.78**
**SEM**	**0.68**	**1.89**

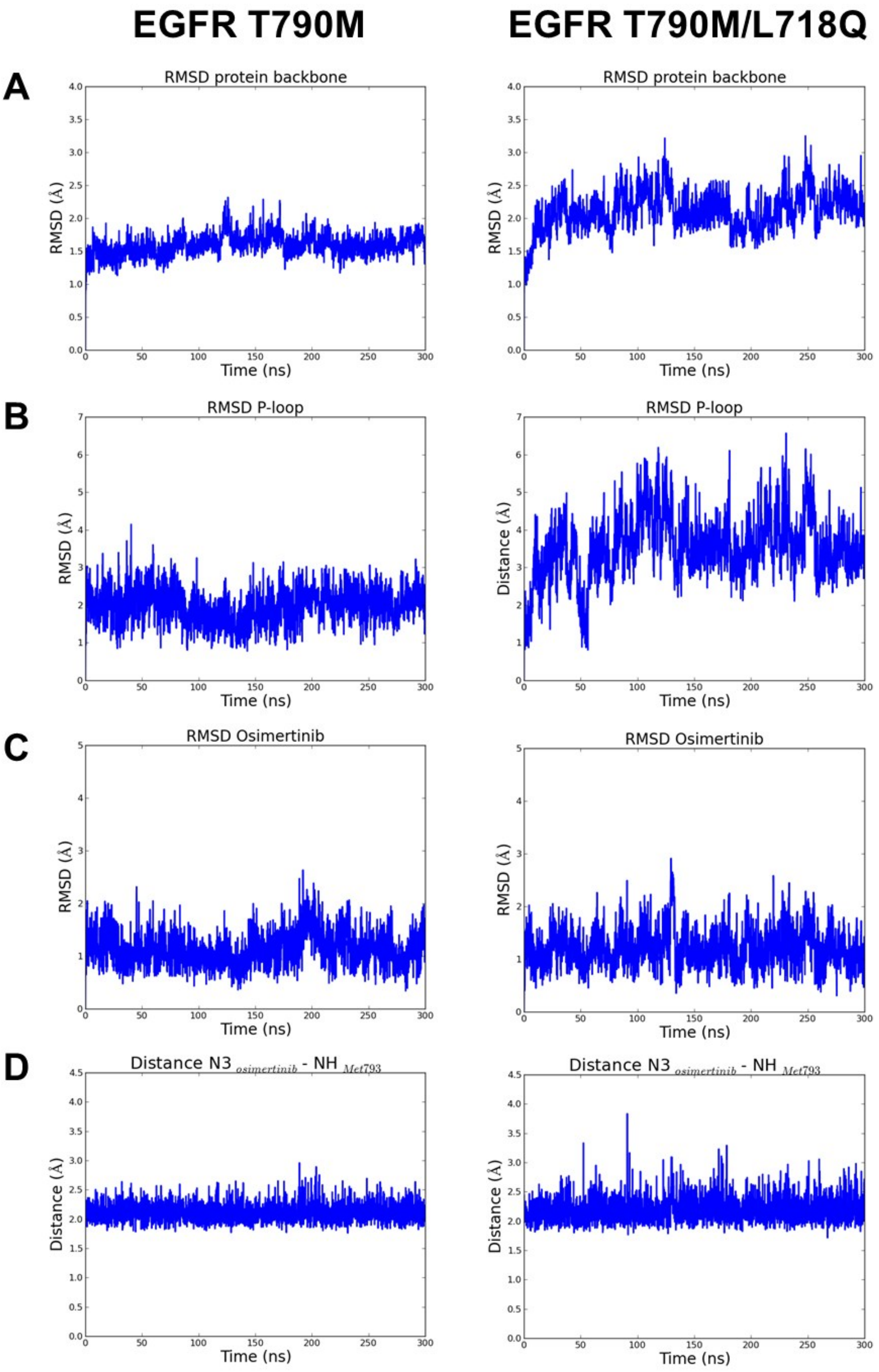

Fig. S3. Analysis of MD trajectory of replica 1. RMSD time series of protein backbone (panel A), RMSD time serie of P-loop backbone (panel B), RMSD time series of osimertinib heavy atoms (panel C), time series of the distance between the N3-pyrimidine nitrogen of osimertinib and backbone N-H group of Met793 (panel D).

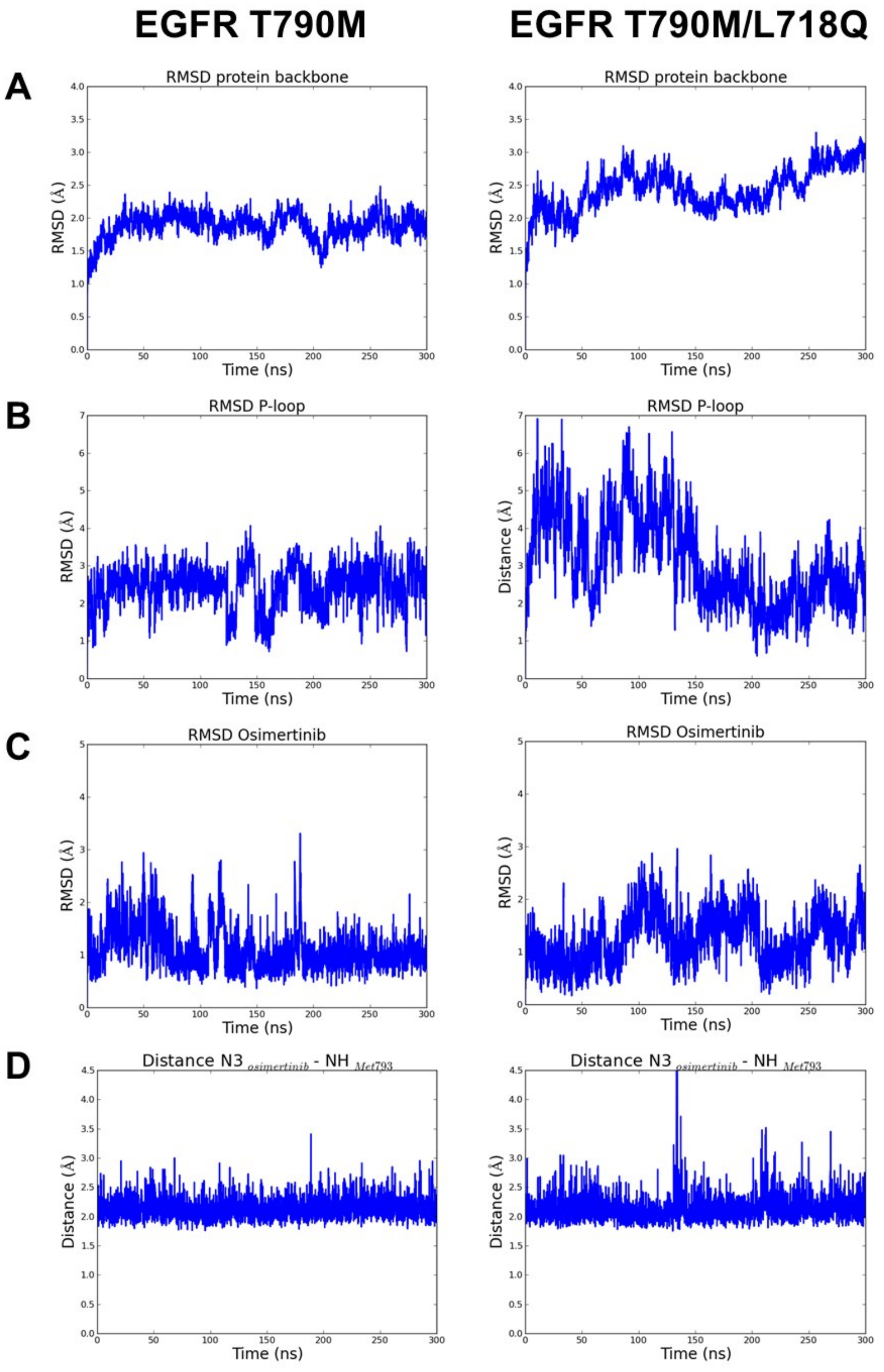

Fig. S4. Analysis of MD trajectory of replica 2. RMSD time series of protein backbone (panel A), RMSD time serie of P-loop backbone (panel B), RMSD time series of osimertinib heavy atoms (panel C), time series of the distance between the N3-pyrimidine nitrogen of osimertinib and backbone N-H group of Met793 (panel D).

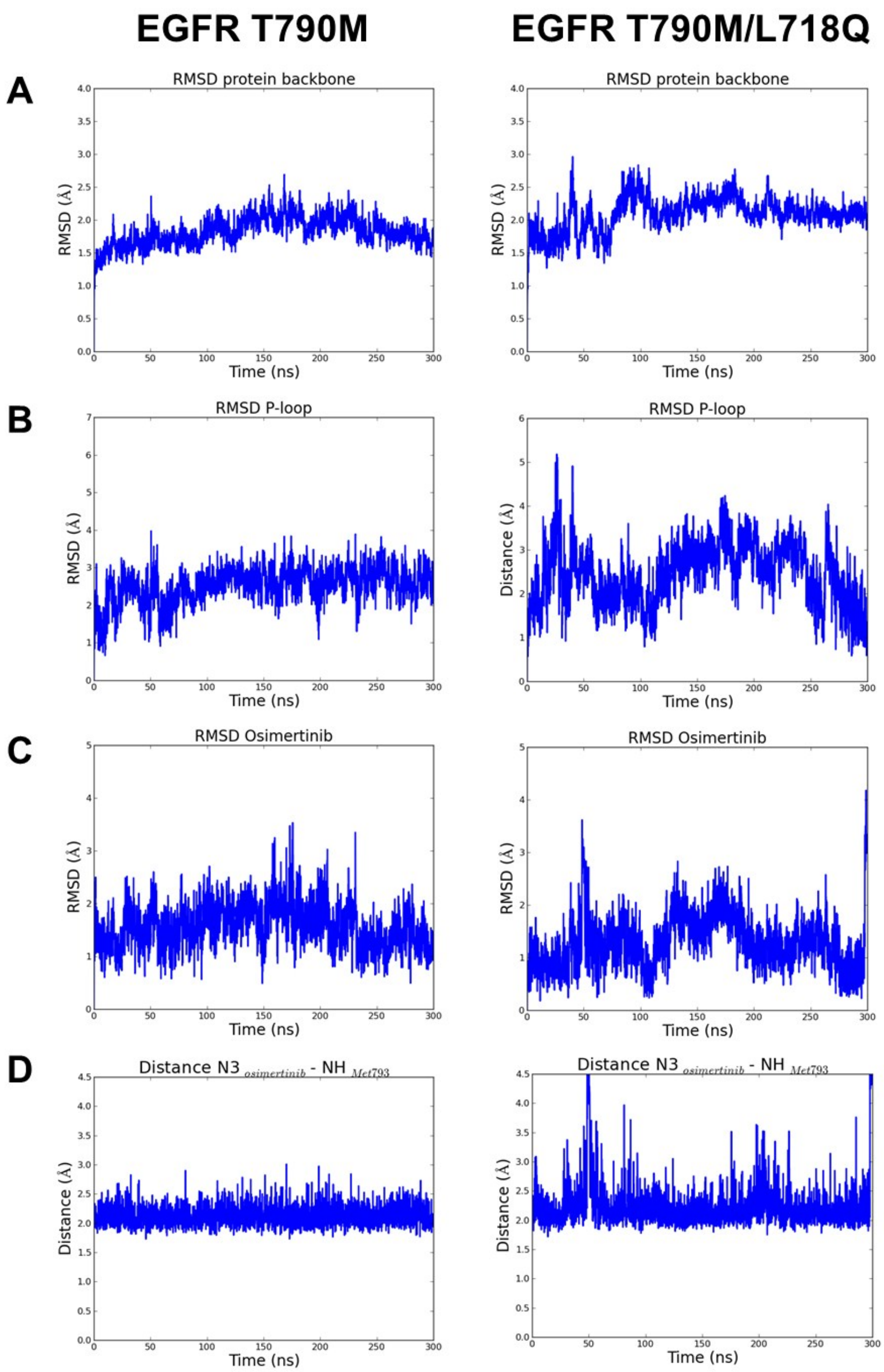

Fig. S5. Analysis of MD trajectory of replica 3. RMSD time series of protein backbone (panel A), RMSD time serie of P-loop backbone (panel B), RMSD time series of osimertinib heavy atoms (panel C), time series of the distance between the N3-pyrimidine nitrogen of osimertinib and backbone N-H group of Met793 (panel D).

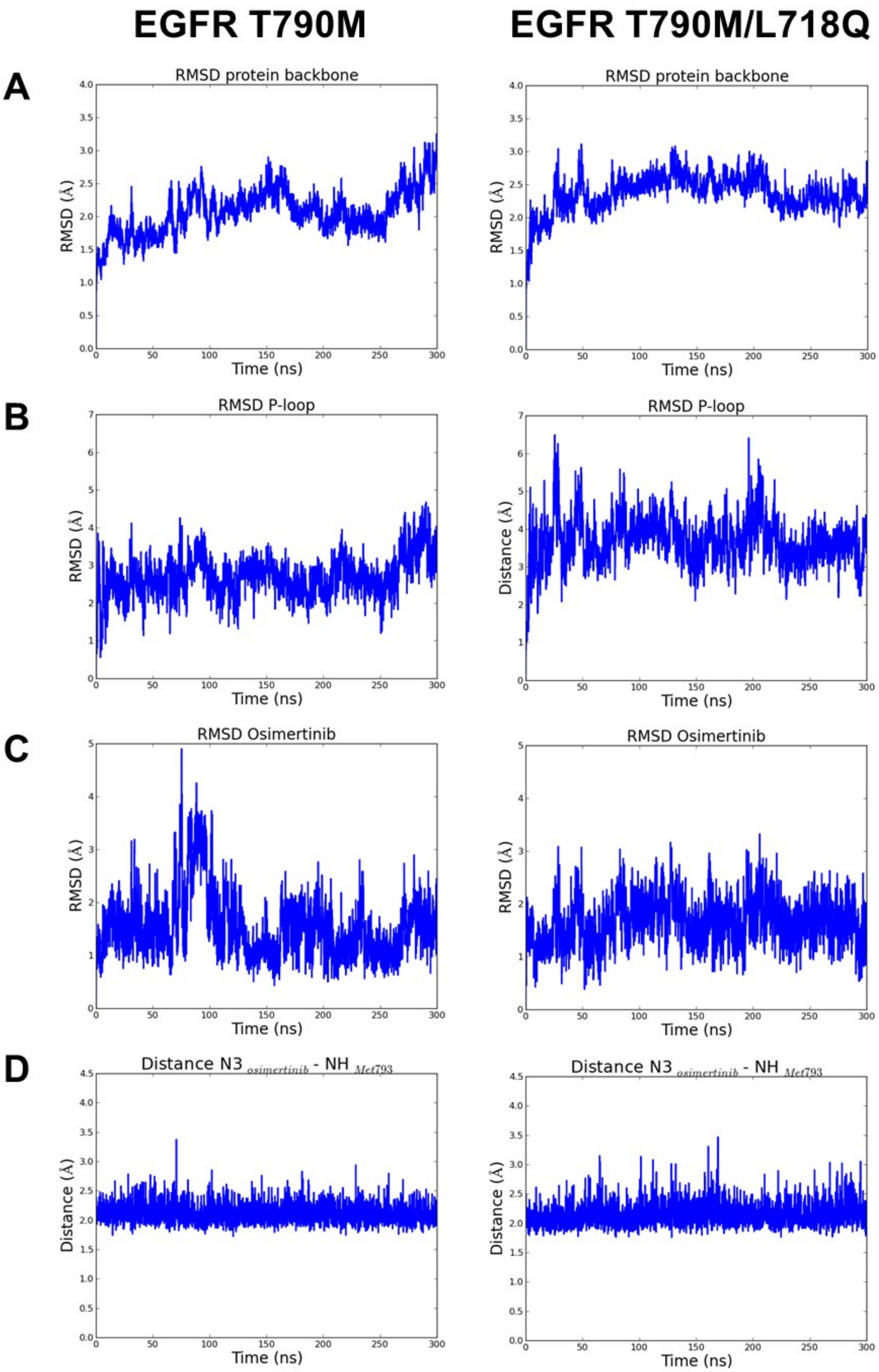

Fig. S6. Analysis of MD trajectory of replica 4. RMSD time series of protein backbone (panel A), RMSD time serie of P-loop backbone (panel B), RMSD time series of osimertinib heavy atoms (panel C), time series of the distance between the N3-pyrimidine nitrogen of osimertinib and backbone N-H group of Met793 (panel D).

Table S4. Fraction of reactive conformations for EGFR T790M and EGFR T790M/L718Q as obtained from trajectory analysis of each independent MD run. The numbers reported in the table represent the percentage of snasphots in which the nucleophile (S_{Cys797}) and electrophile ($C\beta_{acrylamide}$) were separated by a distance smaller than the sum of the Van der Waals radius (i.e. 3.9 Å).

	**EGFR T790M**	**EGFR T790M/L718Q**
**replica1**	20.47%	0.73%
**replica2**	14.37%	0.07%
**replica3**	12.07%	0.07%
**replica4**	5.9%	0.73%

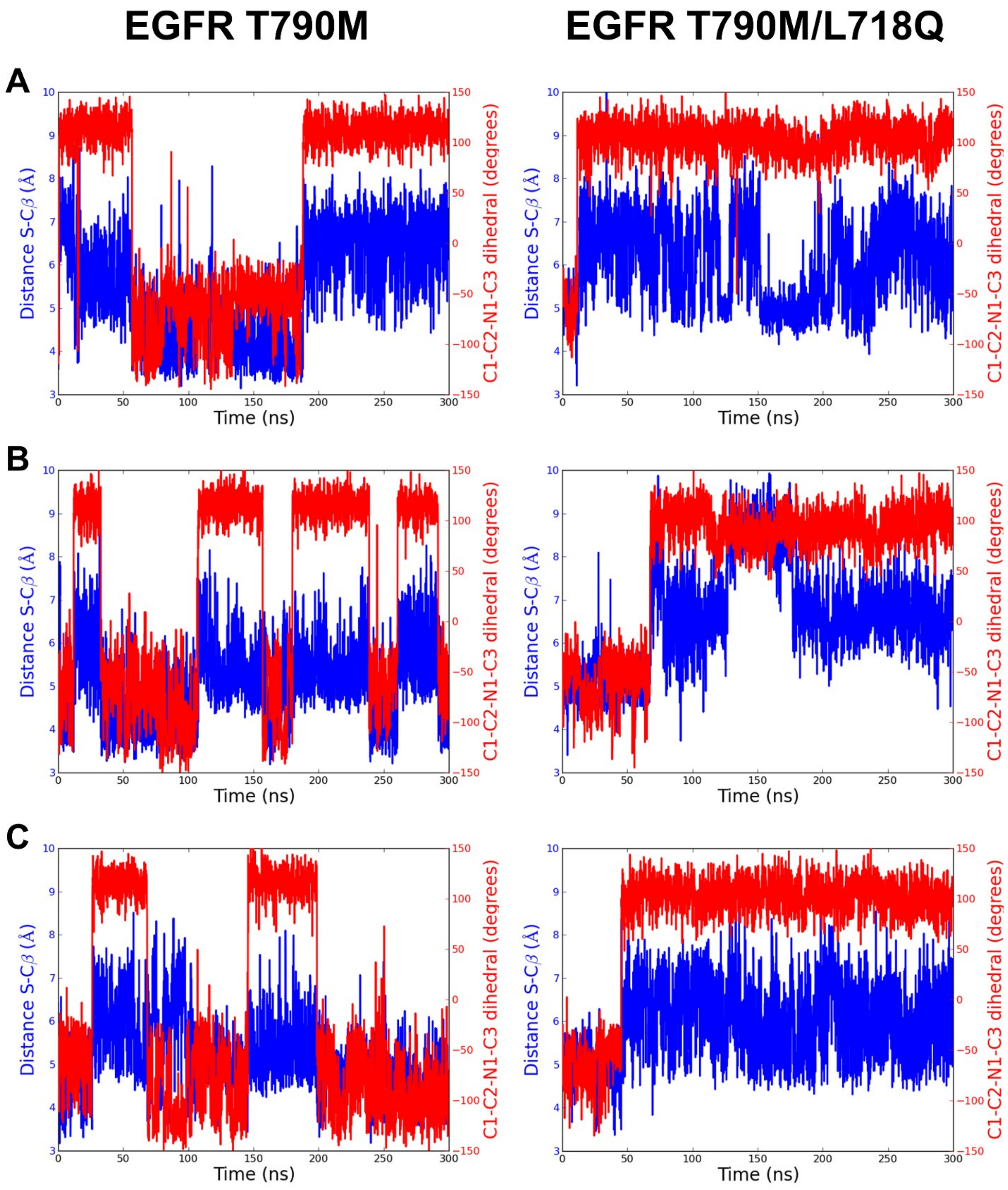

Fig. S7. Time series for the S-Cβ distance (blu line) and the C1-C2-N1-C3 dihedral (red line) for EGFR T790M and EGFR T790M/L718Q in replica 2 (panel A), replica 3 (panel B) and replica 4 (panel C).

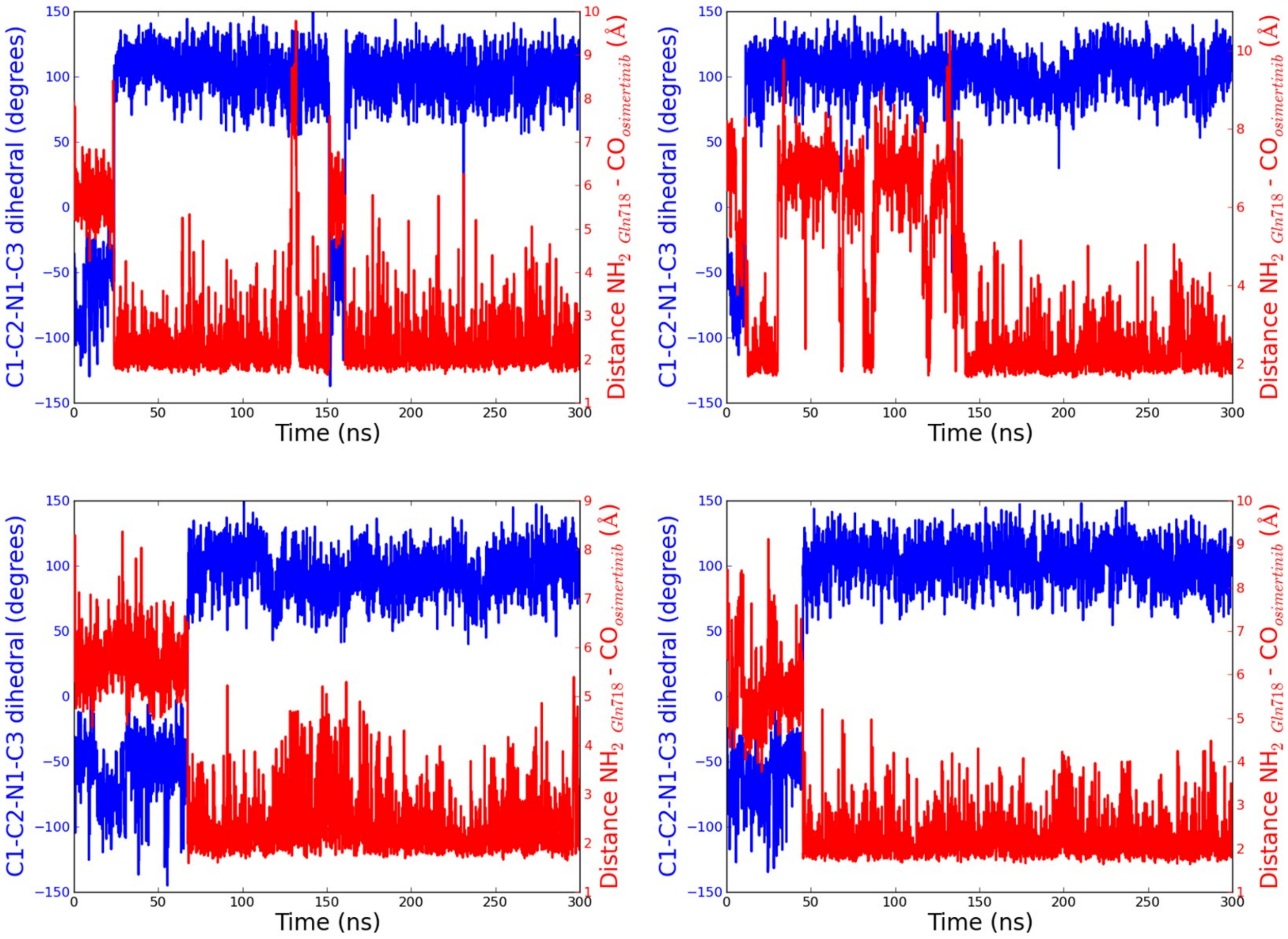

Fig. S8. Time series comparing the evolution of the C1-C2-N1-C3 dihedral (blue line) with the evolution of the H-bond distance undertaken by the polar side chain of Gln718 and the carbonyl oxygen of the acrylamide group of osimertinib (red line) for each of the four MD replicas.

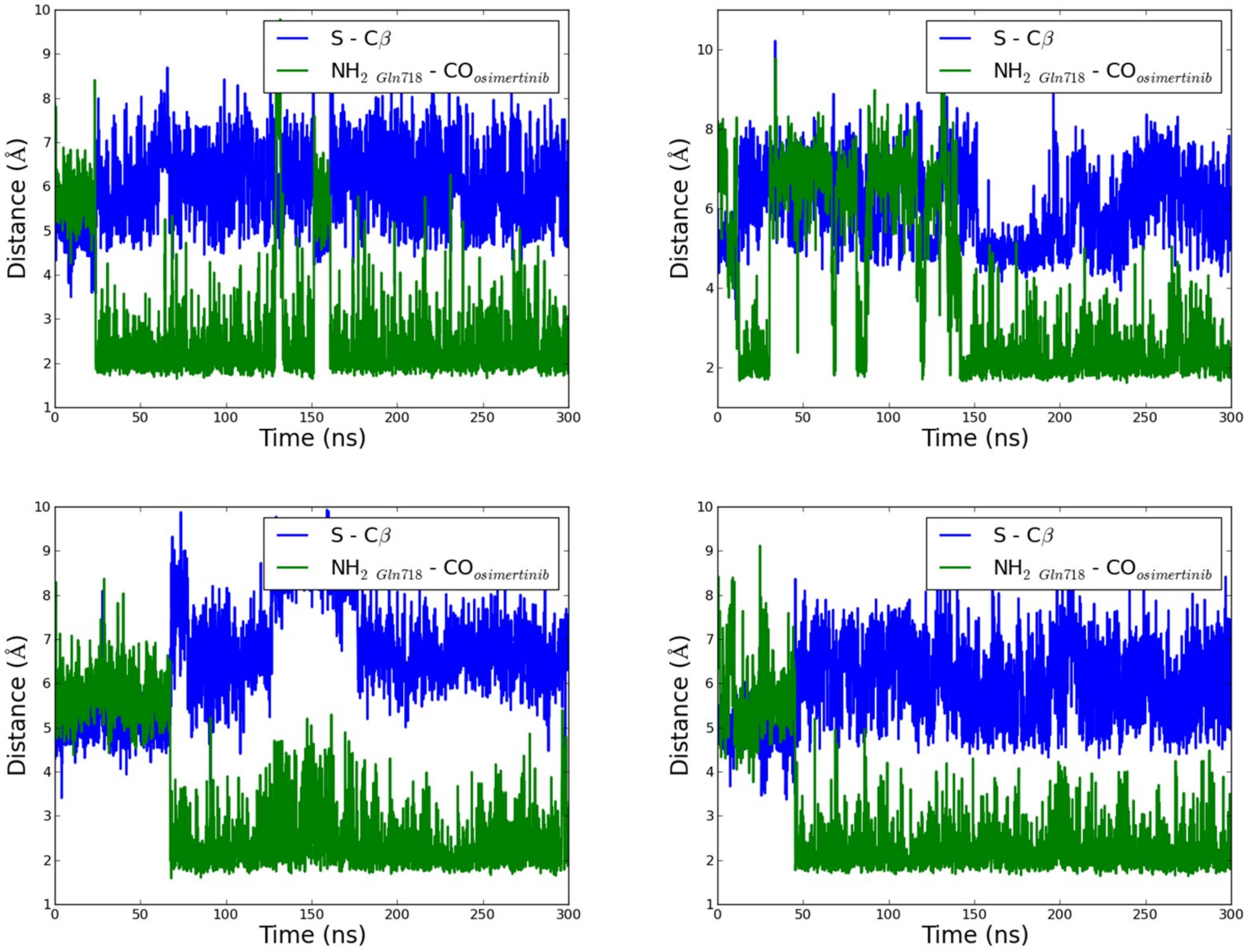

Fig. S9. Time series comparing the evolution of the S-Cβ distance (blue line) with the evolution H-bond distance undertaken by the polar side chain of Gln718 and the carbonyl oxygen of the acrylamide group of osimertinib (green line) for each of the four MD simulations.

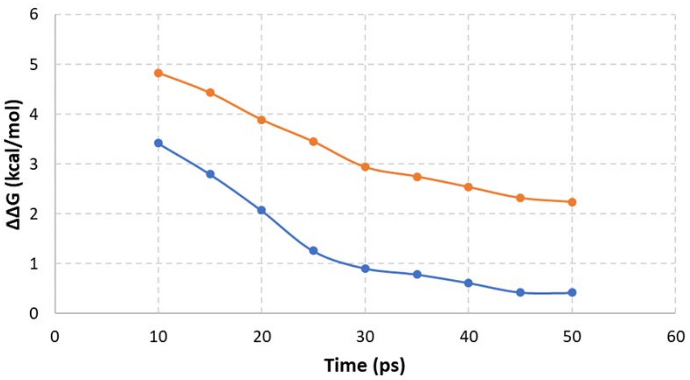

Fig. S10. Convergence of the simulations of Cys797 deprotonation by Asp800 evaluated. the difference in the free-energy (kcal/mol) was calculated between Cys797−S−/Asp800−COOH and Cys797−SH/Asp800−COO− states as function of the time of simulation from data collected every 5ps of simulations for each window.

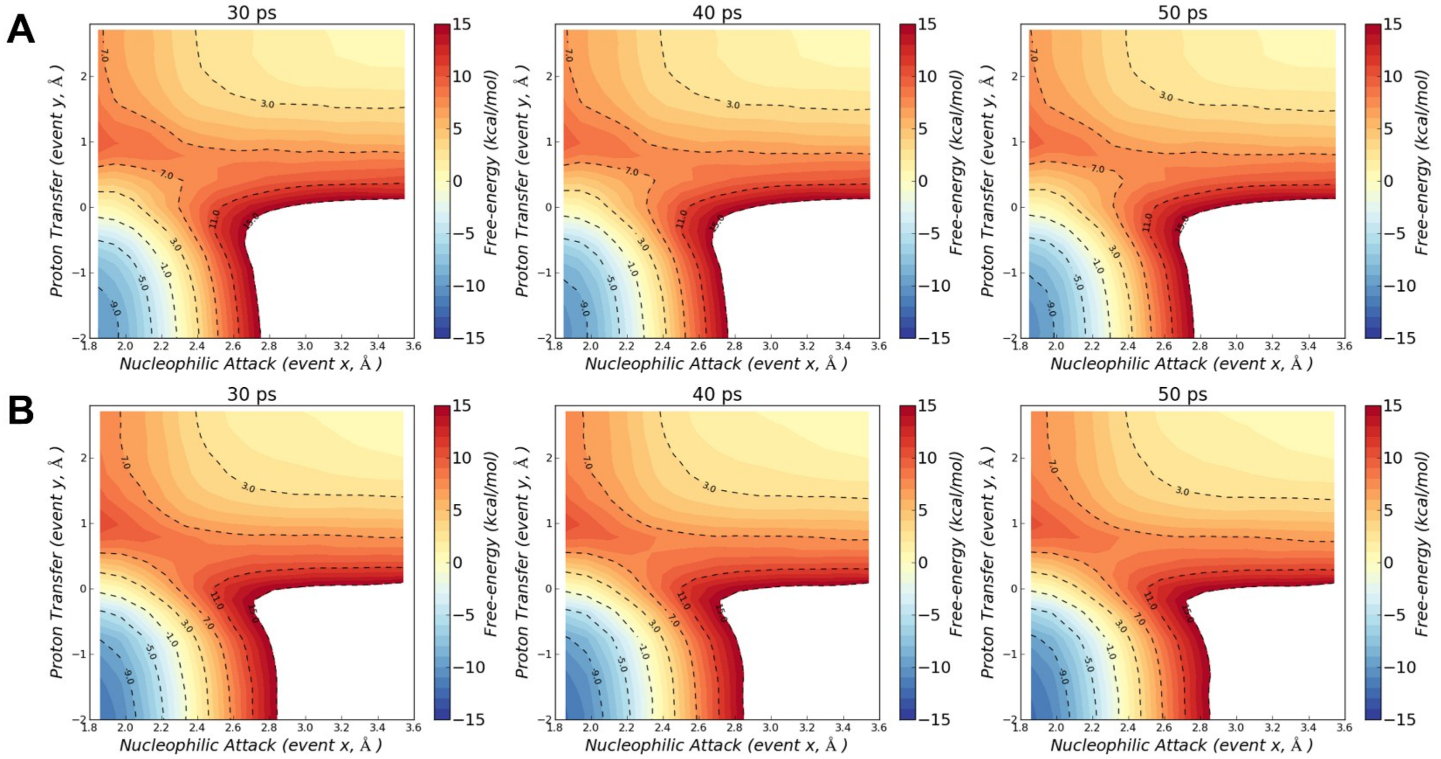

Fig. S11. Convergence of Cys797 alkylation by QM/MM-US simulations. Free energy surface were build from data collected at 30, 40 and 50 ps of simulations for each window for Osimertinib-EGFR T790M complex (panel A) and for Osimertinib-EGFR T790M/L718Q complex (panel B).

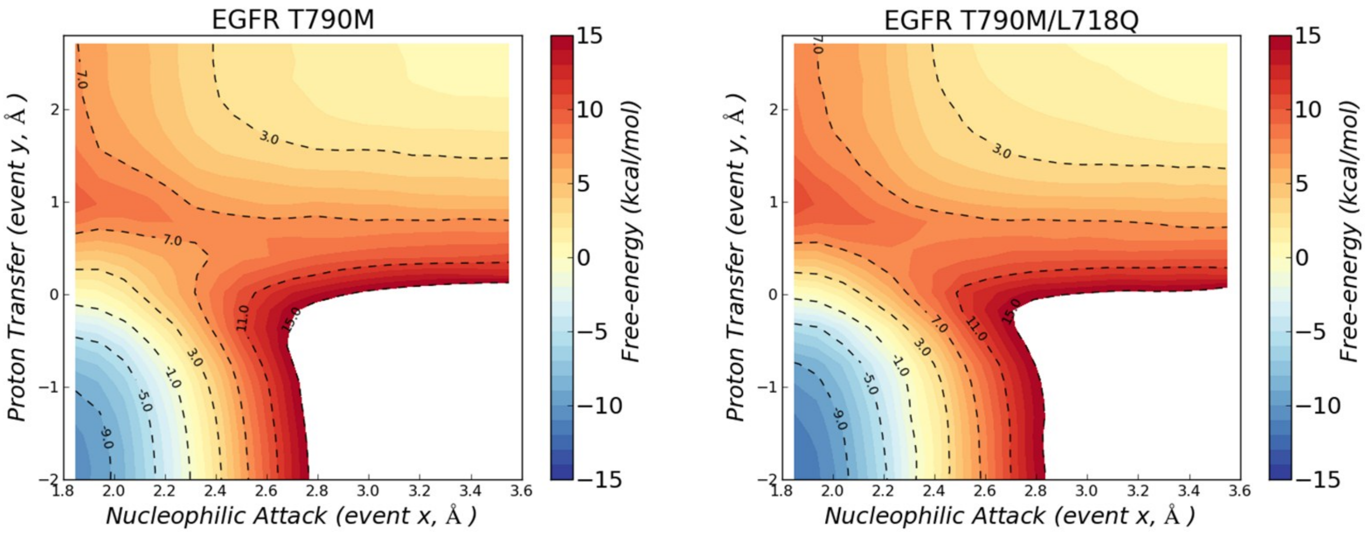

Fig. S12. SCC-DFTB/AMBER FES of the replica of Cys797 alkylation by osimertinib in the presence of EGFR 790M (left panel) or EGFR T790M/L718Q (right panel). The reaction coordinates (nucleophilic attack and proton transfer), are given in angstroms. Free energies are given in kcal/mol, and the contour levels are set at 1 kcal/mol while dashed-contour lines are set every 4 kcal/mol.

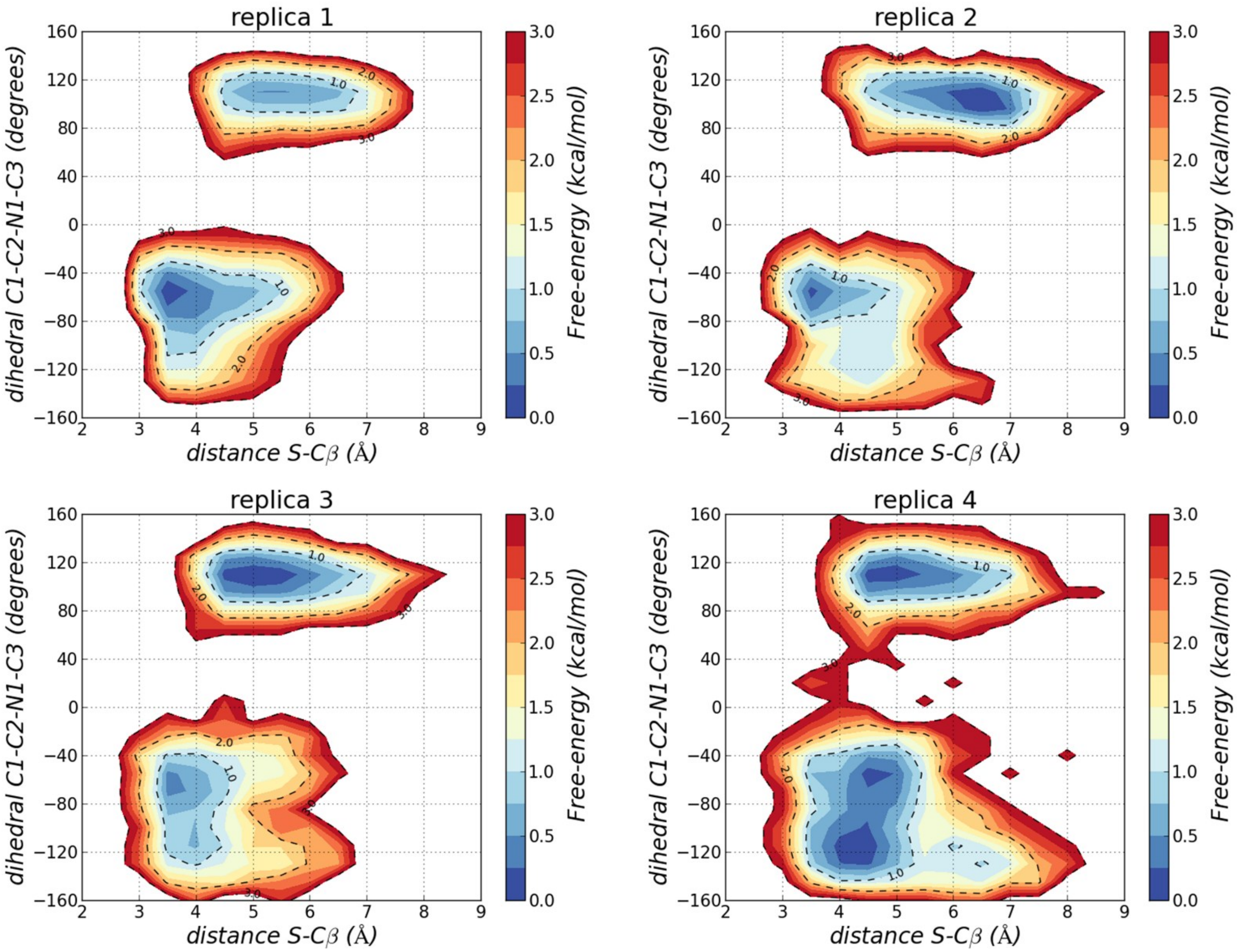

Fig. S13. Free energy surface build from frequency distribution of conformations obtained from each of the four independent 300-ns long MD simulations for EGFR T790M in non-covalent complex with osimertinib. Free energies are given in kcal/mol, the contour levels are set at 0.25 kcal/mol while dashed-contour lines are set every 1 kcal/mol.

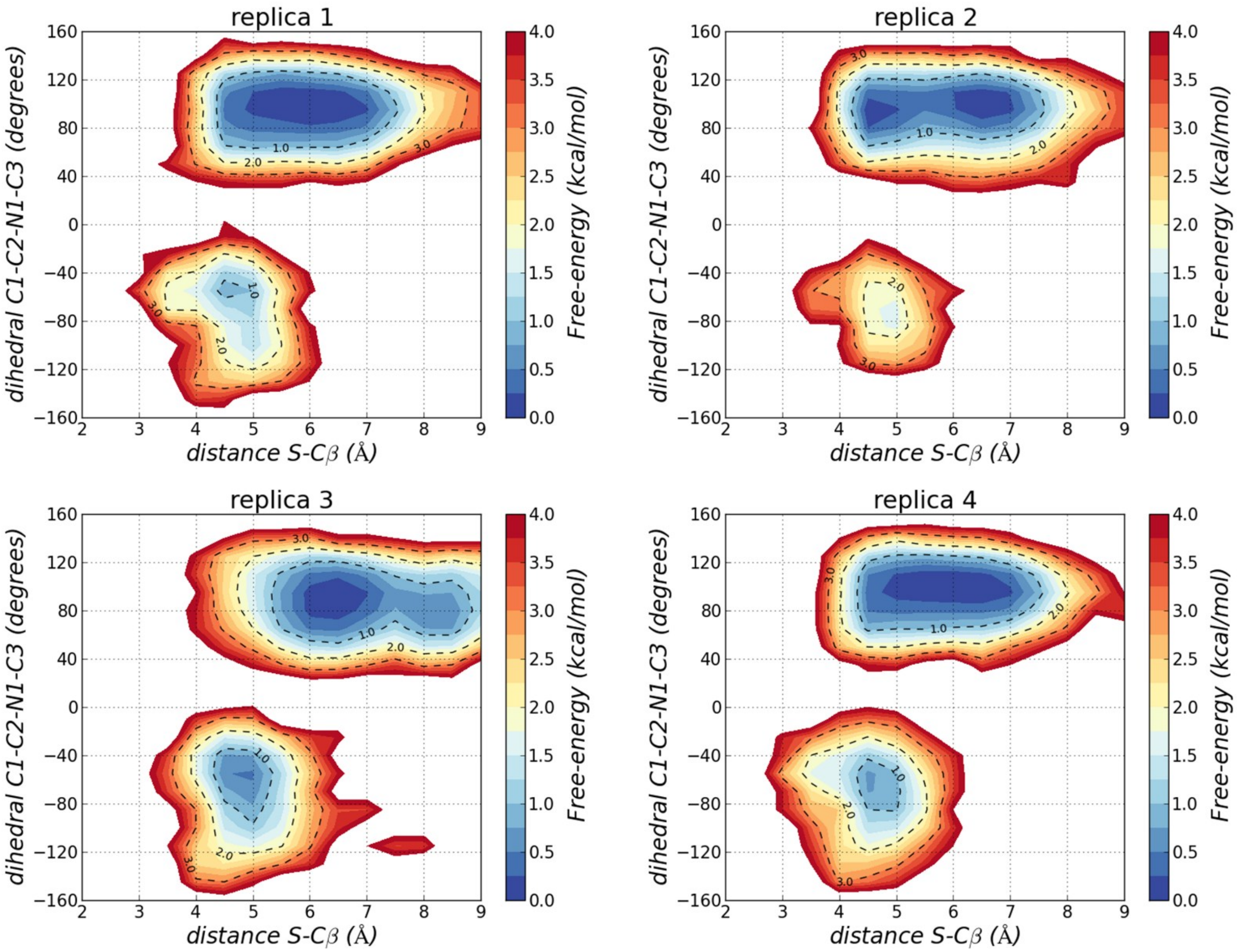

Fig. S14. Free energy surface build from frequency distribution of conformations obtained from four independent 300-ns long MD simulations for EGFR T790M/L718Q in non-covalent complex with osimertinib. Free energies are given in kcal/mol, the contour levels are set at 0.25 kcal/mol while dashed-contour lines are set every 1 kcal/mol.
